# Trends in outpatient healthcare visits among adults aged 50 years and older in 27 European countries: analysis of population-based survey data, 2004–2022

**DOI:** 10.1016/j.lanepe.2025.101407

**Published:** 2025-09-24

**Authors:** Văn Kính Nguyễn, Anna Reuter, Mirna Abd El Aziz, Till Bärnighausen

**Affiliations:** a Heidelberg Institute of Global Health, Medical Faculty and University Hospital, Heidelberg University, Im Neuenheimer Feld 130.3, Heidelberg 69120, Germany; b Bundesinstitut für Bevölkerungsforschung, Friedrich-Ebert-Allee 4, Wiesbaden 65185, Germany; c London School of Hygiene and Tropical Medicine, Keppel Street, London WC1E 7HT, UK

**Keywords:** Healthcare utilisation, Healthcare disruptions, Ageing, Europe

## Abstract

**Background:**

Understanding trends in healthcare utilisation is key to policy and planning, especially after a prolonged pandemic that led to shutdowns of services in many societal sectors. The aim of this study is to provide comprehensive, cross-country evidence on long-term outpatient care trajectories for the older population in Europe.

**Methods:**

We used longitudinal data from 147,116 individuals aged 50 years or older in 27 European countries from the Survey of Health, Ageing and Retirement in Europe (SHARE). We divided the data into three phases: pre-pandemic (2004–2019), pandemic (2021; 2020 excluded due to changes in data collection), and post-pandemic (2022). We used a Bayesian spatiotemporal model to analyse the number of outpatient healthcare visits by sex (male/female) over time and to establish their association with age and chronic conditions.

**Findings:**

From 2004 to 2019, the rate of outpatient healthcare visits for a reference individual (defined as 75 years old with no chronic conditions) varied more than threefold across European countries: For men, visits were lowest in Sweden in 2004, with 1.52 [95% uncertainty interval (UI): 1.45–1.61] visits, and highest in Luxembourg in 2019, with 4.62 [4.30–4.90] visits. For women, visits were also lowest in Sweden in 2004 (1.82 [1.74–1.89]) and highest in Luxembourg in 2019 (5.60 [5.20–5.97]). During COVID-19, healthcare visits of men ranged between 0.06 [0.06–0.07] in Italy and 0.45 [0.41–0.49] in Germany, relative to the rate expected without the pandemic. For women, the relative rate ranged between 0.05 [0.05–0.06] in Italy and 0.42 [0.39–0.45] in Germany. Relative reductions were larger for older adults and those with chronic conditions. After COVID-19, the rates of outpatient healthcare visits mostly returned to pre-pandemic levels, but they remained significantly lower for individuals with cardiovascular disease, diabetes, and chronic lung disease.

**Interpretation:**

We show that outpatient healthcare visits for older adults in most European countries declined substantially during the COVID-19 pandemic, but have since then mostly recovered back to their pre-pandemic levels. An important exception to the general recovery of healthcare visits after the pandemic is people living with chronic conditions, who have continued to utilise healthcare at lower levels than in the pre-pandemic era. Policy innovations are needed to ensure that chronic disease patients re-engage with care after major health systems disruptions.

**Funding:**

Research in this article is a part of the European Union’s H2020 SHARE COVID-19 project (Grant Agreement No. 101015924).

**Research in context**

**Evidence before this study**

We searched PubMed for studies on the trend of healthcare visits using the term (“healthcare”[Title/Abstract] OR “health care”[Title/Abstract] OR “primary care”[Title/Abstract] OR “outpatient”[Title/Abstract]) AND (“visit∗”[Title] OR “utili∗ation”[Title] OR “access”[Title]) AND (population[Title/Abstract] OR survey[Title/Abstract] OR register[Title/Abstract] OR administrative[Title/Abstract] OR review[Title/Abstract]) AND (trend[Title/Abstract] OR change[Title/Abstract] OR “over time”[Title/Abstract] OR COVID[Title/Abstract]), including all studies published between January 1st 2000 and December 31st 2024. Only few studies focused on European countries other than the United Kingdom, and on changes in healthcare utilisation over time. Studies varied vastly in the type of healthcare utilisation examined and the study population. Evidence on the impact of the COVID-19 pandemic on healthcare utilisation beyond 2020 is rare.

**Added value of this study**

Our study is the first to examine harmonised individual, longitudinal data on outpatient healthcare visits among the population aged 50 years or older in Europe, spanning periods before, during and after the COVID-19 pandemic. The data from the Survey of Health, Ageing and Retirement in Europe (SHARE) allows an international comparison across 27 countries, spanning 18 years, including the aftermath of the COVID-19 pandemic. We find large variation in the number of healthcare visits across Europe before the pandemic. While the pandemic affected countries heterogeneously, rates of healthcare visits recovered to pre-pandemic levels in nearly all countries. Healthcare visits among individuals living with chronic conditions (cardiovascular disease, diabetes, chronic lung disease) declined more severely during the pandemic and did not fully recover once the pandemic had passed.

**Implications of all the available evidence**

Healthcare utilisation is a critical indicator to monitor during public health emergencies. Countries need to identify and implement preparatory policies to reduce the impact of future emergencies on healthcare utilisation. In particular, policies need to be in place to ensure that people living with chronic conditions requiring consistent, longitudinal care (such as hypertension, diabetes, and chronic lung disease) re-engage with care as fast as possible following health systems disruptions during an emergency. In general, more research is needed to establish whether the wide range of healthcare utilisation among older adults across European countries is based on underlying differences in healthcare needs, different organisations of the same type of care, or social conventions and norms.

## Introduction

Europe’s population is ageing. As older adults are expected to live increasingly longer life spans, and make up a growing share of the population, their health becomes a central determinant of overall population health.^1^ Over the past decades, the health of the older population in Europe has steadily improved across a wide range of dimensions.^2^, ^3^, ^4^ This progress, however, will not necessarily continue: In some high-income countries, amenable mortality (i.e., deaths that could have been averted given timely utilisation of effective healthcare) among older adults ceased to decline before the pandemic,^5^ as did CVD mortality.^6^ In addition, COVID-19 mortality disproportionally affected older adults.^7^

Tracking healthcare utilisation, in particular, the number of healthcare visits is an important indicator to ensure healthcare access among the older population: With higher age, healthcare visits are expected to increase, even in absence of health problems, as medical guidelines recommend regular screenings based on age.^8^^,^^9^ Similarly, a certain base level of healthcare visits among a population might be beneficial, because general health checks are correlated with increased chronic disease recognition, treatment, and control.^10^ Put in relation to the health burden of a population, the number of healthcare visits can serve as a proxy for universal healthcare coverage.^11^

Trends in healthcare visits over time can signal potential problems of access to care, especially if disaggregated by population groups and health conditions,^12^^,^^13^ and inform planning of healthcare supply. This is especially crucial in the context of large health shocks, such as the COVID-19 pandemic. On the one hand, even relatively resource-rich health systems as in Europe were overwhelmed by the number of intensive care patients and the high infection risk.^14^ On the other hand, individuals stayed absent due to fear of infection, mobility restrictions, or rejection by healthcare providers.^15^ In some cases, this might have relieved health systems from overutilisation in terms of unnecessary consultations and treatments,^16^ particularly in systems with a high coverage of health insurance, as in many European countries. Yet, if diagnoses were delayed or chronic care interrupted, the long-run consequences could burden health systems in the future, as easily treatable conditions progress to major morbidities.

The massive drop in healthcare utilisation, especially for outpatient care, during the early phase of the COVID-19 pandemic has been widely documented.^17^, ^18^, ^19^, ^20^ While some studies indicate larger reductions among patients in generally good health,^17^ others point towards a delay in diagnoses for cardiovascular diseases, diabetes, cancer, and common mental health problems.^21^, ^22^, ^23^ For delayed melanoma diagnoses alone, it is estimated that $7.65 billion additional costs were incurred in Europe.^24^

No prior studies have focused on the long-term time trends of healthcare utilisation of the older population across Europe, spanning the periods before, during, and after the COVID-19 pandemic. In particular, no study previously investigated how healthcare utilisation changed both during and after the pandemic. A few studies examined specific aspects of healthcare utilisation among older adults, such as telehealth and mental health services,^25^ or described healthcare utilisation during the COVID-19 pandemic in single countries (Germany and the UK).^26^, ^27^, ^28^ In addition, there is little evidence on the trajectory of outpatient care in the general population after the initial phase of the pandemic. Evidence from the UK, Germany, Poland, and Finland suggest that healthcare visits among the general population largely recovered to pre-pandemic levels by 2021, but that this recovery failed to occur for particular aspects of healthcare, for example, mental health services in primary care.^29^, ^30^, ^31^, ^32^

We aimed to establish the trajectories of healthcare visits in Europe before, during and after the COVID-19 pandemic among the population aged 50 years or older. This population is likely to have higher need for regular healthcare than younger populations, because both screening and chronic care needs increase with age. For our study, we use the harmonized data from the Survey of Health, Ageing and Retirement in Europe (SHARE). The longitudinal structure of SHARE allows us to estimate trends in healthcare visits before (between 2004 and 2019), during (2021), and after (2022) the COVID-19 pandemic.

## Methods

### Study design

This study used panel data from 27 European countries, collated from SHARE, waves 1, 2, 4, 5, 6, 7, 8, 9 and the second wave of the COVID-19 surveys.^33^, ^34^, ^35^, ^36^, ^37^, ^38^, ^39^, ^40^, ^41^ The overarching goal of SHARE is to understand ageing in Europe; it collects information from the population aged 50 years or older, as well as cohabiting spouses and partners.^42^ The SHARE surveys captured a broad range of self-reported health and socioeconomic information based on questionnaires that are harmonised across countries and time. The topics of the questionnaire include demographics, physical and mental health, healthcare utilisation, behavioral health risks, cognitive function, employment, income, wealth, consumption, housing, social networks, and future expectations.

SHARE was designed based on the US Health and Retirement Study (HRS) in collaboration of initially 11 country teams.^42^ SHARE was coordinated by the Mannheim (later: Munich) Research Institute for the Economics of Ageing (MEA) until 2023, and by the SHARE BERLIN Institute from 2023 onwards. The first wave was conducted in 2004, spanning 11 European countries and Israel (joining in 2005). The following waves were conducted approximately every two years, and over time a further 16 European countries joined SHARE. Data was collected using Computer-Assisted Personal Interviews (CAPI). Questionnaire translation was organised by each country team, supported by a central coordinator, and followed common guidelines based on established procedures such as in the European Social Survey.^42^ The translations were subject to two appraisals by an expert in survey translation and pre-testing.^42^

In March 2020, data collection for wave 8 had to be stopped due to the outbreak of the COVID-19 pandemic. In summer 2020 and summer 2021, two special COVID-19 surveys were conducted over the phone (Computer-Assisted Telephone Interviews, CATI), using different questionnaires, which contained both standard SHARE questions and new questions to elicit information on pandemic-specific research priorities. From October 2021 till December 2022, wave 9 was conducted (with 80% of interviews in 2022), returning back to the previous standard SHARE questionnaire and CAPI-based data collection. In the following, we refer to the COVID-19 surveys as data from the “pandemic” period, and the wave 9 data as data from the “post-pandemic” period.

### Participants

The SHARE survey covered individuals aged 50 years or older (main respondents) whose regular domicile was in a participating country, as well as their cohabiting spouses or partners. Participants were drawn from country-specific sampling frames (mostly population or civil registers), with the aim to retrieve a sample that was representative of the country’s population aged 50 years or older.^43^ Countries differed in their inclusion of individuals in nursing homes due to the differences in the population recording procedures in the different sampling frames. All main respondents became part of the longitudinal sample, and were traced within the respective country when their residence location changed refreshment samples were drawn based on country-specific schedules. Participation rates varied around 50% using the response rate 1 definition of the American Association for Public Opinion Research, which is a conservative measure, as it assumes complete eligibility among all unsuccessful contacts.^44^^,^^45^ This participation rate is comparable to other European household surveys on health.^46^ Retention rates varied around 80%, with higher retention rates for long-term panel members.^45^ Further details on the survey methodology can be found in the SHARE methodological volumes.^47^^,^^48^

### Ethics approval

The SHARE study was approved by the Ethics Committee at the University of Mannheim (waves 1–4; ethics committee decisions on 13.12.2004, 16.12.2008 and 28.6.2010) and by the Ethics Council of the Max-Planck-Society (waves 5–9 and COVID waves; 15.2.2012, 13.6.2012, 19.2.2014, 23.2.2016, 14.6.2018, 29.5.2020 and 8.6.2021). Where required, country-specific ethics committees or institutional review boards approved implementations of SHARE in the participating countries. All study participants provided written informed consent.

### Procedures

#### Outcome

The primary outcome of the current study was the number of outpatient healthcare visits an individual reported in the period prior to the interview. We focused on outpatient healthcare because we were interested in the long-term trends of those components of healthcare that are regular and routine. Healthcare visits were defined as seeing or talking to a medical doctor or qualified nurse. The questionnaire item excluded visits to dentists and hospital stays, which were elicited separately. In the COVID-19 surveys, healthcare visits were recorded as going to a doctor’s office or a medical facility other than a hospital.

The recall period was 12 months (for SHARE waves 1–9) or the time since the last interview (for the second COVID-19 survey). For the second COVID-19 survey, we used the exact date of interview to calculate the person-months contributed by each individual. For the first COVID-19 survey, the recall period was defined as ‘since the COVID-19 outbreak’, which might vary based on the respondent’s perception of the beginning of the outbreak, and was hence excluded from the analysis. For waves 1, 2, 4, and 5, only values up to 98 were allowed as answer. This limitation was lifted for later waves, except for the COVID-19 surveys, which captured healthcare visits with a binary value (yes/no). Details can be found in the Supplementary Material, Supplementary Table S1.

#### Covariates

On the individual level, we included information on participant’s sex (male/female), age (numeric), existing health conditions (yes/no for each condition separately), as well as country and wave. Existing health conditions were captured as self-reported diagnoses according to a pre-specified list. Types of health conditions that were collected only in some waves were excluded from the analysis (see Supplementary Material, Supplementary Table S2).

In addition, we used the following additional country level data to analyse differences across countries: Gross Domestic Product (GDP) per capita in 2019,^49^ government health expenditure as percent of GDP in 2019,^49^ physician density in 2019,^50^ population density in 2019,^49^ cumulative COVID-19 deaths by June 2021,^51^ and the average Oxford COVID-19 Government Response Tracker (OxCGRT) stringency index till June 2021.^51^

### Statistical analysis

For our analysis, we excluded data on respondents who were staying at a nursing facility at the time of interview (to mirror the exclusion in some of the sampling frames), those younger than 50 years of age at the time of the interview (because our sampling target were individuals aged 50 years or older), and data on respondents aged 100 years or older (due to the very small number of participants in this age range). Ireland participated in only one survey and was not included in this analysis. We focused on European countries only (excluding Israel). Based on 159,288 main respondents in the SHARE data (153,502 excluding Ireland and Israel), data from 147,116 individuals were included in the analyses, resulting in a total of 505,607 observations (Supplementary Material, Supplementary Table S3).

We implemented a combined likelihoods approach to estimate the covariate effect without the influence of pandemic and post-pandemic data, as well as providing a concrete reference for the estimation of the pandemic effects. First, the number of healthcare visits during a time period was modelled as a rate per person-month. This allows different periods of recall to be modelled and projected. We constructed a Bayesian hierarchical model for the number of healthcare visits $y_{ijt}$ in the corresponding recall period $E_{ijt}$ (measured in months) for an individual *i* in country *j* and calendar year *t* as follows:

(1) $$y_{ijt}∼Poisson\;\left(\lambda_{ijt}\right)$$

(2) $$\lambda_{ijt}=\gamma_{ijt}\;E_{ijt}\;\exp\left(\eta_{ijt}\right)$$

In particular, we modelled the number of healthcare visits as a Poisson count that was extended to allow a variance of this count larger than its mean via an overdispersion parameter (*γ*) with a unit prior mean. We modelled the number of healthcare visits separately for women and men. For the pre-pandemic data period, the linear predictor is modelled as

(3) $$\eta_{ijt}=\beta_{0}\;+\;\phi_{j}\;+\;\delta_{j}t\;+\;X_{ijt}\beta_{x}\;+\;f\left(age_{ijt}\right)\;+\;\epsilon_{ijt}$$

where we assumed a country-specific random effect, $\phi_{j}∼N\left(0,\sigma_{j}^{2}\right)$, that varied around the global mean $\beta_{0}$. We included binary indicators for each of the health conditions listed in Table 1 and estimated the independent effect of each condition, denoted as $X_{ijt}\beta_{x}$. A shared effect of the continuous age variable was modelled with a second-order random walk to allow for potential structured nonlinear effects, denoted by $f\left(age_{ijt}\right)$. Trends over time, represented by the calendar year of the interview, were modelled with a random slope term $\delta_{j}$, which allows for differences in the trends among the countries. We assumed that the trends among the countries might be correlated and modelled the slopes with the spatially correlated Besag, York, and Mollié (BYM) model.^52^

**Table 1:** Summary statistics.

Characteristic	Individuals (n = 147,116)	Observations (n = 505,607)
Sex		
Female	81,042 (55%)	285,256 (56%)
Male	66,074 (45%)	220,351 (44%)
Age^a^	63 (56–72)	68 (61–75)
Health conditions^a^		
Heart attack	30,129 (20%)	62,539 (12%)
Hypertension	77,439 (53%)	214,972 (43%)
High cholesterol	52,480 (36%)	123,992 (25%)
Stroke	11,069 (7.5%)	19,979 (4.0%)
Diabetes	25,769 (18%)	66,965 (13%)
Chronic lung disease	15,140 (10%)	30,324 (6.0%)
Cancer	14,559 (9.9%)	25,419 (5.0%)
Stomach/duodenal/peptic ulcer	12,906 (8.8%)	21,227 (4.2%)
Parkinson	2161 (1.5%)	4109 (0.8%)
Cataracts	23,851 (16%)	42,135 (8.3%)
Hip/femoral fracture	5908 (4.0%)	9060 (1.8%)
None	51,396 (35%)	109,334 (22%)
Country		
Austria	7186 (4.9%)	26,391 (5.2%)
Belgium	10,402 (7.1%)	38,761 (7.7%)
Switzerland	4740 (3.2%)	21,068 (4.2%)
Germany	10,425 (7.1%)	32,251 (6.4%)
Denmark	5890 (4.0%)	24,410 (4.8%)
Spain	9131 (6.2%)	31,458 (6.2%)
France	8833 (6.0%)	31,436 (6.2%)
Greece	6520 (4.4%)	25,654 (5.1%)
Italy	8423 (5.7%)	33,871 (6.7%)
Netherlands	6459 (4.4%)	17,274 (3.4%)
Sweden	6742 (4.6%)	25,638 (5.1%)
Czech Republic	9237 (6.3%)	31,992 (6.3%)
Poland	7821 (5.3%)	21,645 (4.3%)
Estonia	8380 (5.7%)	36,581 (7.2%)
Hungary	3797 (2.6%)	8304 (1.6%)
Portugal	2688 (1.8%)	7280 (1.4%)
Slovenia	6760 (4.6%)	25,066 (5.0%)
Luxembourg	2125 (1.4%)	7506 (1.5%)
Croatia	5543 (3.8%)	13,372 (2.6%)
Bulgaria	1959 (1.3%)	4992 (1.0%)
Cyprus	1298 (0.9%)	3414 (0.7%)
Finland	2649 (1.8%)	7063 (1.4%)
Lithuania	2107 (1.4%)	7106 (1.4%)
Latvia	2498 (1.7%)	5719 (1.1%)
Malta	1285 (0.9%)	4289 (0.8%)
Romania	2168 (1.5%)	7329 (1.4%)
Slovakia	2050 (1.4%)	5737 (1.1%)

Data are n (%), median (IQR), or n/N (%).

a For individuals, age is reported as median age at survey entry, and health conditions as ever diagnosed over the survey period.

Individual identification across the waves was modelled with a normally distributed term $\epsilon_{ijt}\;∼\;N\left(0,\sigma_{e}^{2}\right)$ to take into account within-individual variability. We used only the data before the COVID-19 pandemic to inform the model parameters at this step. We tested for beta convergence by regressing the yearly growth rate in the number of healthcare visits on the initial number of healthcare visits.

We calculated and reported the rate of healthcare visits for a reference person before the pandemic (t = 2019) for each included country, defined as an individual with average age effect ($f\left(age_{iju}\right)\approx 0$) and without any of the reported health conditions listed in Table 1 ($X_{ijt}\beta_{x}=0$). This allows for cross-country comparisons net of population differences in age structure and prevalence of diagnosed conditions.

For the pandemic period, the estimated linear predictor from the pre-pandemic model was carried over to provide the reference point for the case without pandemic. The data of this period provides estimates of the changes compared to what would be predicted given the same individual characteristics. The linear predictor for this period of time *u* reads as

(4) $$\begin{array}{c} \eta_{iju}={\hat{\eta }}_{ijt}\;+\;X_{iju}\rho_{xu}\beta_{xt}\;+\;f\left(age_{iju}\right)\;+\;\epsilon_{iju}\;+\;\nu_{ju} \end{array}$$

where ${\hat{\eta }}_{ijt}$ denotes the expected number of healthcare visits without the pandemic; $\rho_{xu}$ models the relative change of a comorbidity effect compared to the pre-pandemic period; a separate age pattern during the pandemic was modelled with the RW2 term $f\left(age_{iju}\right)$; and $\nu_{ju}$ denotes the country-specific change in the mean number of healthcare visits during the pandemic. Similarly, for the post-pandemic period, we assumed that a pandemic carry-over effect might exist and thus replaced the coefficients in Equation (4) with those representing the estimates from wave 9. All equations were jointly estimated, using an augmented data approach and three likelihoods for the three data periods, in which the parameters were copied over to the pandemic and post-pandemic periods, including their uncertainty in the estimates.^53^

SHARE used three approaches to record our primary outcome data, each of which yields a slightly different data likelihood. For the survey waves 1, 2, 4, and 5, SHARE recorded the number of healthcare visits truncated at the value 98; during waves 6–9, SHARE recorded the number of healthcare visits without truncation; during the second COVID-19 survey, SHARE recorded healthcare visits as a binary response, which we interpreted as a Poisson variable truncated at one. The three data recording approaches can be modelled as a censored Poisson at 98, a Poisson, and a censored Poisson at one, respectively. The associated likelihoods read as

(5) $$\begin{array}{c} L=\prod_{ij}\;P\;\left(Y^{d=0}=y_{ij}\right)\prod P\;\left(Y^{d=1}\geq T_{w}\right) \end{array}$$

where d=0,1 is an indicator variable specifying whether the observation was censored and $T_{w}=\left\{1,98\right\}$ denotes the two cases of censored data described above. The model parameters were estimated with the R integrated nested Laplace approximation (INLA) package for approximate Bayesian inference.^54^ We computed posterior medians and 95% credible intervals of our outcomes, including the annual number of healthcare visits, the differential of the time trend, and the effect of COVID-19, using posterior simulations of 1000 parameter samples. The models were compared using the Watanabe-Akaike Information Criterion (WAIC). Computations were done using the High-Performance Computing (HPC) cluster at the Center for Scientific Computing (CSC) of Goethe University Frankfurt, with a single model runtime of ∼26 h on 40-cores 128 Gb RAM AlmaLinux.

### Role of the funding source

The funder had no role in data analysis, data interpretation, writing of the report, or decision to publish.

## Results

### Sample characteristics

As shown in Table 1, 55% of all respondents were female, representing 56% of all observations across waves. The median age at time of the first interview was 63 (interquartile range [IQR]: 56–72), while the median age over all interviews was 68 (IQR: 61–75). Over the course of the observation period, more than half of the respondents (53%) were diagnosed with hypertension at some point in time, resulting in 43% of observations with a hypertension diagnosis across waves. This is followed by high cholesterol (ever reported by 36% of all individuals, present in 25% of all observations), heart attacks (20%, 12%), diabetes (18%, 13%), cataracts (16%, 8.3%), chronic lung disease (10%, 6.0%), cancer (9.9%, 5.0%), stomach/duodenal/peptic ulcer (8.8%, 4.2%), stroke (7.5%, 4.0%), hip/femoral fracture (4.0%, 1.8%), and Parkinson (1.5%, 0.8%). About one third (35%) of respondents reported no diagnosed health condition at any point during the observation period (22% of observations).

### Healthcare visits before the pandemic

**Fig. 1:**
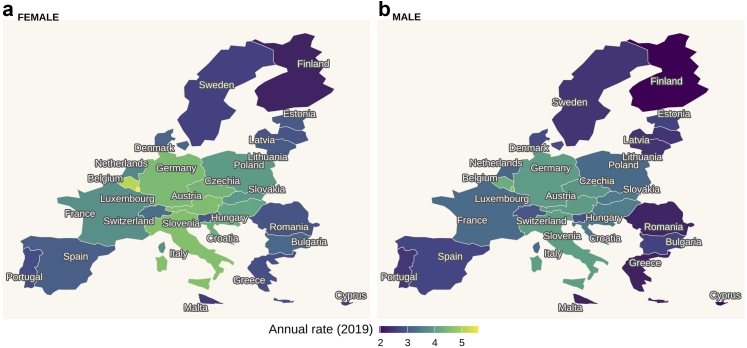
**Rate of outpatient healthcare visits (annualised for the year 2019 for (a) females and (b) males).** The posterior estimates are conditioned on the reference group in the regression model: no comorbidity and 75 years of age.

Fig. 1 shows the expected annual number of outpatient healthcare visits by country and sex estimated for the year 2019, controlling for age, health conditions, and trends over time. The numbers were calculated by setting effects of each health condition and age effect included in the model to near zero, corresponding to a reference individual without any of the health conditions and 75 years old. The rates varied substantially across countries, and was higher for women than for men. The highest rates of outpatient healthcare visits in 2019 were observed in the centre of Europe, with 5.60 (95% uncertainty interval (UI): [5.20–5.97]) visits for women and 4.62 [4.30–4.90] visits for men in Luxembourg, followed by Belgium (women: 5.10 [4.94–5.29], men: 4.26 [4.08–4.41]), Italy (women: 4.58 [4.44–4.71], men: 4.00 [3.82–4.15]), Austria (women: 4.54 [4.41–4.80], men: 3.97 [3.80–4.20]), and Germany (women: 4.47 [4.33–4.62], men: 3.98 [3.81–4.13]). The lowest rates of visits in 2019 were observed in Northern Europe and small islands, with 2.20 [2.07–2.34] visits for women and 1.96 [1.84–2.12] visits for men in Finland, followed by Cyprus (women: 2.61 [2.46–2.78], men: 2.02 [1.87–2.23]), and Malta (women: 2.52 [2.35–2.68], men: 2.18 [2.01–2.37]). The lowest rates of visits over the observation period were observed in 2004 in Sweden, with 1.52 [1.45–1.61] visits for men and 1.82 [1.74–1.89] visits for women.

Across Europe, there was a large variation in trends in healthcare visits over time, with a slight increase overall (Supplementary Fig. S1). There was a small convergence, with countries with initially higher rates of healthcare visits experiencing a smaller increase than countries with initially lower rates (beta convergence for men: −2.34% [95% UI: −4.99% to 0.31%], beta convergence for women: −2.4% [−4.36% to −0.36%]). Within most countries, trends were relatively similar for women and men, while for some countries, such as Italy, Spain, France, and Czechia, the gender gap in healthcare visits narrowed over time.

### Pandemic shock and recovery

**Fig. 2:**
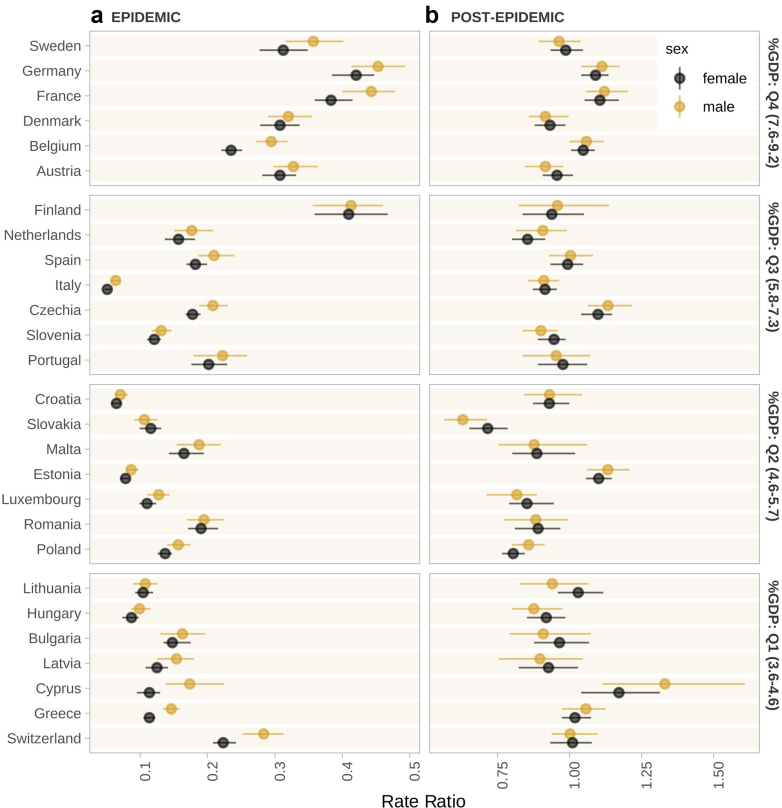
**Reduction of healthcare visits (a) during and (b) after the COVID-19 pandemic.** The figure shows the posterior estimates of the percentage reductions in the rate of outpatient healthcare visits compared to the expected value based on the past time trends. The points are the posterior medians of the percentage reduction; the lines are the 95% uncertainty intervals. The countries are grouped by domestic general government health expenditure as percent of gross domestic product (GDP) for visualisation purposes (right-hand side vertical axis).

Fig. 2 shows the estimated relative reduction of the rate of outpatient healthcare visits during and after the COVID-19 pandemic by country. The estimates were derived from the difference between the expected rate if the pandemic had not occurred, based on the pre-pandemic data. Individuals in all countries experienced a large drop in the rate of healthcare visits (Fig. 2); the least affected country was Germany, where the rate of healthcare visits was 0.45 [95% UI: 0.41–0.49] relative to the expected number of healthcare visits for men and 0.42 [0.39–0.45] for women. The most affected countries were Italy (women: 0.06 [0.05–0.06], men: 0.06 [0.06–0.07]), Croatia (women: 0.07 [0.06–0.07], men: 0.07 [0.06–0.08]), Estonia (women: 0.08 [0.07–0.08], men: 0.09 [0.08–0.10]), and Hungary (women: 0.09 [0.08–0.10], men: 0.10 [0.08–0.12]), where the healthcare visits came nearly to a halt. In 2022, healthcare visits returned back to the pre-pandemic levels for most of the countries, with the rate of healthcare visits between 0.90 and 1.10 compared to pre-pandemic levels. The rate still remained low in Slovakia (men: 0.64 [0.56–0.71], women: 0.71 [0.64–0.79]), Luxembourg (men: 0.81 [0.74–0.89], women: 0.85 [0.78–0.94]), and Poland (men: 0.85 [0.80–0.91], women: 0.80 [0.77–0.84]), and was relatively high compared to pre-pandemic levels in Cyprus (men: 1.36 [1.18–1.59], women: 1.15 [1.02–1.30]).

We grouped the countries according to domestic government health expenditure as percent of GDP in 2019. Countries with very high health expenditure tended to have a smaller decline of healthcare visits during COVID-19, compared to those with a lower health expenditure. Overall, a 1 percentage point increase in government health expenditure as percent of GDP in 2019 was correlated with a 0.05 higher rate of healthcare visits during the pandemic compared to pre-pandemic levels in both women [95% UI: 0.03–0.06] and men [0.03–0.07]. Neither the pandemic reduction nor the post-pandemic recovery was associated with the severity of the pandemic (measured as either cumulative deaths from COVID-19 or the average OxCGRT stringency index by the time of the second COVID-19 survey), the population density in 2019, or the physician density in 2019. If Luxembourg, as an extreme outlier, was omitted, there was a negative correlation between the pandemic decline and per-capita GDP in 2019.

### Healthcare visits across age and comorbidities

**Fig. 3:**
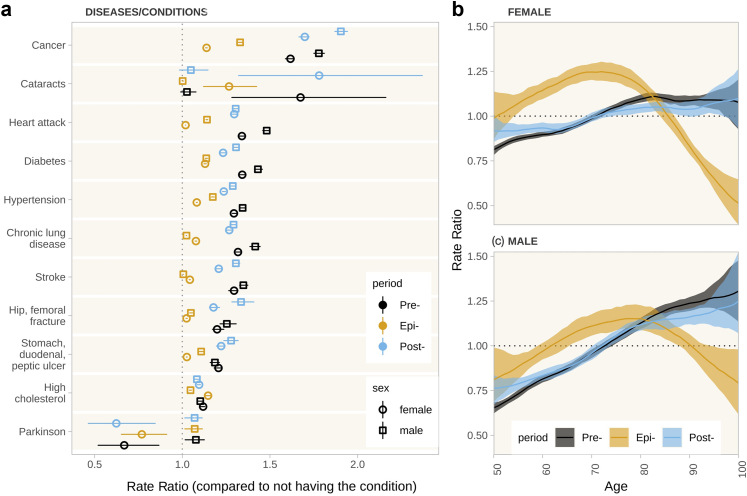
**Rate ratio of healthcare visits (a) by diseases/conditions, (b) for females by age, and (c) for males by age.** The point/line and line range/shaded colors denote the posterior median and 95% uncertainty interval, respectively. The diseases and health conditions were compared against not being diagnosed with the respective condition. Age was modelled as nonlinear terms for each of the periods (Pre = pre-pandemic, Epi = during the pandemic, Post = post-pandemic) separately.

Fig. 3 shows that most chronic conditions were associated with a higher rate of outpatient healthcare visits. The largest difference in the rate of healthcare visits was among people with cancer compared to people without cancer. Having chronic lung diseases, diabetes, or a history of stroke or heart attack was associated with an increase in the visit rate by approximately 20% compared to those without the disease. For most health conditions, the association was larger for men than for women. For stroke, stomach and related ulcers and high cholesterol, the association was similarly high for both sexes. During the pandemic period, the number of healthcare visits decreased relatively more for most of the chronic conditions, but remained slightly higher than for respondents without the respective condition. After the pandemic, the association with the number of healthcare visits remained significantly lower for men with a prior heart attack (relative risk post-pandemic 1.30 [95% UI: 1.29–1.31], pre-pandemic 1.48 [1.46–1.50]), hypertension (post: 1.29 [1.28–1.30], pre: 1.34 [1.33–1.36]), diabetes (post: 1.31 [1.29–1.32], pre: 1.43 [1.41–1.46]), and chronic lung disease (post: 1.29 [1.27–1.31], pre: 1.42 [1.39–1.44]), but was significantly higher for men with a cancer diagnosis (post: 1.90 [1.87–1.94], pre: 1.78 [1.75–1.81]) and stomach, duodenal, or peptic ulcers (post: 1.28 [1.24–1.32], pre: 1.19 [1.16–1.21]). For women, the picture was similar, except for ulcers: The association remained significantly lower for women with a prior heart attack (post: 1.30 [1.28–1.31], pre: 1.34 [1.32–1.36]), hypertension (post: 1.24 [1.23−1.25], pre: 1.29 [1.28–1.31]), stroke (post: 1.21 [1.19–1.22], pre: 1.30 [1.26–1.32]), diabetes (post: 1.23 [1.22–1.25], pre: 1.34 [1.32–1.36]), and chronic lung disease (post: 1.27 [1.25–1.29], pre: 1.32 [1.30–1.34]), but was significantly higher for cancer (post: 1.70 [1.67–1.72], pre: 1.62 [1.59–1.64]).

During the pre-pandemic period, the number of visits increased with age, with a steeper trend for men compared to women. This pattern, however, changed during the pandemic into an inverse u-shaped relationship: the number of healthcare visits increased linearly with age for respondents aged 50–75 years but decreased for respondents at higher ages. After the pandemic, the positive linear trend returned for both sexes, although with slightly flatter slopes, particularly for women.

## Discussion

Evidence on the trajectories of outpatient healthcare visits is scarce for most European countries. We analysed SHARE, a harmonised, longitudinal dataset of individuals aged 50 years or older across 27 European countries and found large cross-country variation in the rate of outpatient healthcare visits. Based on the time trends in these visits prior to the COVID-19 pandemic (between 2004 and 2019), we estimated a large decline in the number of healthcare visits due to the pandemic. While the extent of the decline differed by country, age group, and prior diagnoses, the major share of the decline was reduced quickly after 2021. An important exception to this general post-pandemic recovery were people with prior chronic disease diagnoses (cardiovascular disease, diabetes, and chronic lung disease), who continued to utilise outpatient healthcare at lower rates than pre-pandemic.

The pre-pandemic, positive age gradient in healthcare visits is in line with previous findings on the population aged 50 years or older in Europe.^13^^,^^55^ The correlation is likely driven by the accumulation of healthcare needs as people age.^56^ In addition, age proxies time to death and the age gradient in healthcare visits might therefore also reflect the fact that many people utilise healthcare most extensively shortly before their deaths.^57^ Finally, many clinical guidelines recommend increased healthcare utilisation with increasing age, e.g., for routine screening and follow-up visits for chronic conditions, which may be partially responsible for the observed age gradient.

We found that during the pandemic, individuals above 75 reduced their healthcare visits more than proportionally, such that the age gradient reversed. An important focus of policies to prepare for future pandemics and similar crises should thus be systems that ensure that the very old safely receive the healthcare they need. Such policies could include new models of home and care home visits, age-dependent healthcare prioritisation schemes, and rapid and intensified information campaigns on changes in healthcare availability for the very old. A study of the COVID-19 surveys in SHARE found that self-perceived unmet need for healthcare utilisation due to COVID-19 declined with age.^15^ This discrepancy with our results could be explained by the fact that the questions to measure self-perceived unmet need used in these surveys did not include many of the reasons for missed visits due to COVID-19, which older adults were plausibly more likely to experience than younger adults, such as changes in the location of healthcare, lack of public transport, and difficulties in navigating new medical appointment systems.

We show that the age gradient of outpatient healthcare utilisation returned to a linear, albeit flatter, form after the pandemic. The relative increase in outpatient healthcare utilisation after the pandemic among the young (50–60 years of age) could represent a catch-up effect on a backlog in general health checks and screenings, which were foregone during the pandemic because of their elective nature. Indeed, many clinical guidelines recommend routine health checks and screenings in this age range.^8^^,^^9^ Findings from England, where routine blood pressure screening rates in this age group decreased during the pandemic, support this explanation.^58^

Previous studies with SHARE data analysed the number of diagnoses or indices based on diagnosed conditions. These studies found a positive association between multimorbidity and healthcare visits.^55^^,^^59^^,^^60^ We confirmed this overall association and showed that the difference in healthcare visits among diagnosed and undiagnosed respondents was particularly high for cancer, and comparatively low for stomach ulcers and high cholesterol levels. During COVID-19, the associations decreased, resulting in a relatively larger gap in care for people with chronic conditions. There was no clear pattern of this decrease according to the type of condition. In particular, we did not observe systematically larger decreases for people suffering from those conditions that put people at high risk of complications from COVID-19 infection.

The number of healthcare visits increased more than proportionally for individuals with cancer after COVID-19, possibly indicating that their conditions might have become more severe. This result is similar to findings from the UK, where attendance volume decreased for cancer patients during the lockdowns, followed by large increases in the consultation rate afterwards.^61^ At the same time, the number of healthcare visits increased less than proportionally for people living with chronic conditions such as cardiovascular disease, diabetes, and chronic lung disease. As a result, patients with these chronic conditions still experienced a gap in their care compared to the time before the pandemic, which likely implies that many people no longer received needed treatment monitoring and calibration. Without further attention to ensure full reengagement in care, these chronic conditions are thus likely to worsen after the pandemic and induce higher healthcare costs in the medium and long run.

Most European countries, as many countries worldwide, reacted to the onset of the pandemic with sudden and strict lockdown policies.^51^ Yet, European countries differed widely in their pandemic preparedness, pandemic management, and the timing and intensity of large outbreaks and excess mortality.^62^^,^^63^ In a prior study, health expenditure and GDP per capita, but not the average stringency index or population density, were associated with excess mortality.^62^ We find the same assocations for the number of healthcare visits. Despite these systematic cross-country differences in the changes in healthcare utilisation during the pandemic, in most countries utilisation returned to prepandemic levels.

Our study has important limitations in several categories of research functions. The first category is interpretation. The number of healthcare visits measures healthcare utilisation (directly) and healthcare access (indirectly), but does not allow judgement on whether utilisation is objectively needed. As we lack data on the number of objectively needed healthcare visits for those individuals participating in SHARE, we cannot distinguish between underutilisation, adequate utilisation, and overutilisation. Hence, a higher rate of healthcare visits does not necessarily reflect that healthcare needs are better met. Still, differences in the rate of healthcare visits across groups and over time hint at potential healthcare deficits for one of the groups, and thus indicate the need for further investigation and potential policy intervention. The second category of limitations relates to our measurement approach. As healthcare visits in SHARE are self-reported, we cannot rule out potential reporting biases common to survey data. Particularly, the comparatively long recall period of 12 months might lead to an under-reporting of healthcare visits^64^ and thus an underestimation of actual utilisation. According to a rigorous synthesis of problems in measuring healthcare utilisation using self-reports, very old participants may be most prone to biased recall, suggesting that we should be most cautious in interpreting the data from our oldest study participants.^64^ Still, our results on differences in utilisation across time and groups will hold, as long as recall bias does not systematically differ across these dimensions of comparison. Relatedly, the exclusion of individuals at nursing facilities might lead to an underestimation of the rate of healthcare visits among the very old population, because individuals living in nursing facilities are likely to be on average in worse health than those living at home, and thus require more care. Another limitation is the change in the measurement of healthcare utilisation during COVID-19. Firstly, only the extensive margin, i.e., whether a doctor was visited, was assessed during the pandemic. Secondly, the phrasing of the question eliciting outpatient healthcare utilisation was changed: During COVID-19, respondents were asked whether they went to a facility other than a hospital; before and after the pandemic, respondents were asked whether they have seen or talked to a doctor or nurse. We tried to incorporate these differences by modelling different Poisson distributions, but cannot rule out that the change in measurement approach led to systematic differences in our main outcome, which may not be fully captured by the model. However, given that wave 9 returned to the previous questionnaire, we are confident that the reported post-COVID changes accurately reflect the difference to the pre-COVID period. Finally, our analyses are based on household survey data, which might be subject to non-response bias due to systematic differences in response rates across groups. Previous analyses of SHARE suggest that response rates do not differ in major ways by sex and age,^43^ and that there is no clear attrition pattern regarding socioeconomic characteristics.^65^ However, we cannot completely rule out that the SHARE sample is selective with regards to socioeconomic status or health.

Overall, our findings show that after the COVID-19 pandemic outpatient healthcare utilisation largely returned to pre-pandemic levels for the population aged 50 years or older across Europe. People with chronic noncancer conditions, however, continued to utilise healthcare at lower levels than before the pandemic. Policy innovations are needed to ensure that older adults living with chronic conditions in Europe reengage with their care following the COVID-19 pandemic. To prepare for future pandemics and similar public health crises, systems that support older adults in their routine healthcare when access to physical care is restricted should be designed and tested.

## Contributors

AR and TB conceptualised the study. VKN, AR, and TB developed the methods. VKN and AR accessed and verified the data as provided by SHARE. VKN analysed the data. VKN and AR wrote the first draft of the manuscript. All authors reviewed and edited the final draft. All authors had full access to all the data in the study and had final responsibility for the decision to submit for publication.

## Data sharing statement

Access to the SHARE data can be applied for with the SHARE Research Data Center (https://share-eric.eu/data). Code will be made available at https://github.com/kklot/SHARE.

## Editor note

The Lancet Group takes a neutral position with respect to territorial claims in published maps and institutional affiliations.

## Declaration of interests

AR benefitted from the European Union’s H2020 SHARE COVID-19 project (Grant Agreement No. 101015924) and the Horizone Europe END-VOC project (Grant Agreement No. 101046314), with all payments made to the institution. VKN benefitted from funds from the German Federal Ministry of Education and Research (BMBF) Network of University Medicine 2.0: “NUM 2.0”, Grant No. 01KX2121, Coverchild Programme (Grant No. D10021866), Collpan Programme (Grant No. D10021865), with all payments made to the institution. TB reports scientific research grants from the European Union (Horizon Europe and Horizon 2020), US National Institutes of Health, Wellcome, Germaan National Research Foundation, German Ministry of Education and Research, Bill & Melinda Gates Foundation, Fleming Fund, UNAIDS, Health + Life Alliance Heidelberg-Mannheim, Alexander von Humboldt Foundation, International Vaccine Institute, Else Kröner Fresenius Foundation, League of European Research Universities (LERU), German Corporation of International Cooperation (GIZ), Volkswagen Foundation, German Development Bank (KfW), African Academy of Sciences, and European and Developing Countries Clinical Trials Partnership, and received support for attending meetings and/or travel by WHO, Disease Control Priorities Project 4, Peking Union Medical College (PUMC), Baden-Württemberg Foundation, and Africa Health Research Institute (AHRI), with all payments made to the institution. TB is Editor-in-Chief at PLoS Medicine (payments received personally), Chair at the International Scientific Advisory Board of the EU Horizon grant “HIGH Horizons—Heat Indicators for Global Health Monitoring, Early Warning Systems and health facility interventions for pregnant and postpartum women, infants and young children and health workers” (unpaid), Member of the Scientific Advisory Board at Leibniz Research Network INFECTIONS (LFV) (unpaid), and the Virchow Foundation Berlin (unpaid). MAEA declares no conflict of interest.
