# Supplementary material for: Trends in outpatient healthcare visits among adults aged 50 years and older in 27 European countries: analysis of population-based survey data, 2004–2022
Source: Lancet Reg Health Eur. 2025 Sep 24;57:101407. doi: 10.1016/j.lanepe.2025.101407 (PMC12541641; doi:10.1016/j.lanepe.2025.101407)
Supplement: Supplementary Fig. S1 and Tables S1–S7 [file mmc1.pdf]

Supplementary Material

Table of contents

Survey questions 2
Sample size 4
Pre-pandemic trends in NHV 5
Detailed estimates 6

Survey questions

Table S1: Survey items on number of healthcare visits

Wave	Question	Variable
1	Please think about your care during the last twelve months. Since {month & last year}, about how many times in total have you seen or talked to a medical doctor or qualified nurse about your health? Please exclude dentist visits and hospital stays, but include emergency room or outpatient clinic visits.	hc002_
2,4,5	Now please think about the last 12 months. About how many times in total have you seen or talked to a medical doctor or qualified nurse about your health? Please exclude dentist visits and hospital stays, but include emergency room or outpatient clinic visits.	hc002_
6,7	Now please think about the last 12 months. Since {month & last year} about how many times in total have you seen or talked to a medical doctor or qualified/registered nurse about your health? Please exclude dentist visits and hospital stays, but include emergency room or outpatient clinic visits.	hc602_
8,9	During the last 12 months, that is since {month & last year}, about how many times in total have you seen or talked to a medical doctor or qualified/registered nurse about your health? Please exclude dentist visits and hospital stays, but include emergency room or outpatient clinic visits.	hc602_
COVID II	Since {last interview}, did you go to a doctor's office or a medical facility other than a hospital?	caq120_

Note: For the second COVID-19 data collection wave, mean imputation was used for the time of last visit if the individual was not interviewed in first COVID-19 data collection wave.

Table S2: Survey item on health conditions.

Answer options	Wave 1	2	4	5	6	7	8	9
A heart attack including myocardial infarction or coronary thrombosis or any other heart problem including congestive heart failure	x	x	x	x	x	x	x	x
High blood pressure or hypertension	x	x	x	x	x	x	x	x
High blood cholesterol	x	x	x	x	x	x	x	x
A stroke or cerebral vascular disease	x	x	x	x	x	x	x	x
Diabetes or high blood sugar	x	x	x	x	x	x	x	x
Chronic lung disease such as chronic bronchitis or emphysema	x	x	x	x	x	x	x	x
Asthma	x	x						
Arthritis, including osteoarthritis, or rheumatism	x	x	x					
Osteoporosis	x	x						
Cancer or malignant tumour, including leukaemia or lymphoma, but excluding minor skin cancers	x	x	x	x	x	x	x	x
Stomach or duodenal ulcer, peptic ulcer	x	x	x	x	x	x	x	x
Parkinson disease	x	x	x	x	x	x	x	x
Cataracts	x	x	x	x	x	x	x	x
Hip fracture or femoral fracture	x	x	x	x	x	x	x	x
Other fractures		x	x	x	x	x	x	x
Alzheimer's disease, dementia, organic brain syndrome, senility or any other serious memory impairment		x	x	x	x	x	x	x
Benign tumor (fibroma, polypus, angioma)		x						
Other affective or emotional disorders, including anxiety, nervous or psychiatric problems				x	x	x	x	x
Rheumatoid Arthritis				x	x	x	x	x
Osteoarthritis, or other rheumatism				x	x	x	x	x
Chronic kidney disease					x	x	x	x
None	x	x	x	x	x	x	x	x
Other conditions, not yet mentioned	x	x	x	x	x	x	x	x

Note: In wave 1, the question text was "Has a doctor ever told you that you had any of the conditions on this card? Please tell me the number or numbers of the conditions." In all subsequent waves, the question text was "[Has a doctor ever told you that you had/Do you currently have] any of the conditions on this card? [With this we mean that a doctor has told you that you have this condition, and that you are either currently being treated for or bothered by this condition.] Please tell me the number or numbers of the conditions." Only conditions assessed in all regular waves were included in the analysis.

Sample size

Table S3: Exclusion criteria and sample size

Exclusion steps	Remaining sample size
All cases excluding wave 3 (SHARELIFE)	569,835
Excluding Israel and Ireland	532,478
Excluding those living in nursing homes	528,469
Excluding those age<50 or age>100	521,637
Excluding those with missing information on health care visits	506,945
Excluding those with implausible education information	506,920
Excluding those with missing information on physical health conditions	505,607

Note: Information on age and sex was complete. Missing information on other characteristics led to the exclusion of 16,030 cases, representing 3% of the sample after all other exclusion steps were applied.

Pre-pandemic trends in NHV

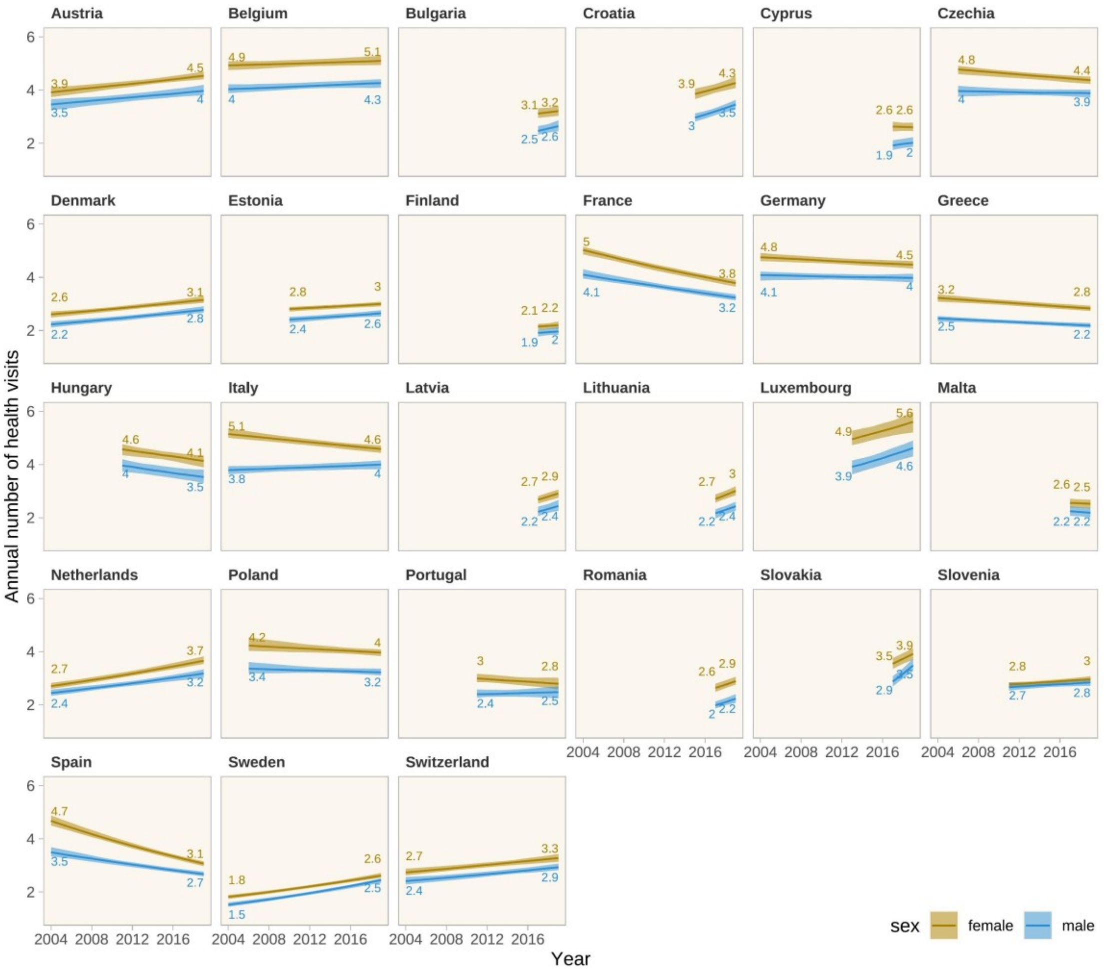

Figure S1: Posterior estimate of the annual rate of healthcare visits over time from 2004 to 2019. The line and its color shade are the posterior median and 95% uncertainty interval. The estimates are conditioned on the reference group in the regression model: no comorbidities, an average age (approx. 75).

Detailed estimates

Table S4: Estimated NHV by country and sex from 2004-2019

Year	Country	Sex	2.5% Quant.	Median	97.5% Quant.
2004	Austria	male	3.244199	3.460106	3.662921
2005	Austria	male	3.284774	3.494889	3.680657
2006	Austria	male	3.325859	3.530927	3.704924
2007	Austria	male	3.379521	3.564859	3.721738
2008	Austria	male	3.425780	3.601352	3.740787
2009	Austria	male	3.464312	3.635793	3.764271
2010	Austria	male	3.503291	3.667981	3.795868
2011	Austria	male	3.551597	3.698551	3.832392
2012	Austria	male	3.594402	3.729973	3.870946
2013	Austria	male	3.633689	3.765922	3.905992
2014	Austria	male	3.665187	3.799657	3.947811
2015	Austria	male	3.694690	3.828295	3.994102
2016	Austria	male	3.730863	3.860150	4.041881
2017	Austria	male	3.762956	3.899215	4.082895
2018	Austria	male	3.782890	3.937422	4.138312
2019	Austria	male	3.800858	3.970442	4.201758
2020	Austria	male	3.823528	4.011040	4.259771
2021	Austria	male	3.847863	4.045841	4.321583
2022	Austria	male	3.867877	4.084259	4.384417
2017	Bulgaria	male	2.324126	2.464562	2.646342
2018	Bulgaria	male	2.404520	2.554225	2.722185
2019	Bulgaria	male	2.452132	2.643808	2.863810
2020	Bulgaria	male	2.481193	2.733460	3.012805
2021	Bulgaria	male	2.498934	2.817244	3.185286
2022	Bulgaria	male	2.516650	2.904888	3.395005
2004	Belgium	male	3.883873	4.034797	4.216610
2005	Belgium	male	3.909189	4.049865	4.215877
2006	Belgium	male	3.931470	4.060354	4.220124
2007	Belgium	male	3.952495	4.074560	4.233116
2008	Belgium	male	3.973634	4.088359	4.241866
2009	Belgium	male	3.995887	4.105081	4.249627
2010	Belgium	male	4.020443	4.123086	4.258893
2011	Belgium	male	4.046720	4.139696	4.272074
2012	Belgium	male	4.063808	4.154753	4.291558

(Continued on Next Page...)

Table S4: Estimated NHV by country and sex from 2004-2019 ***(continued)***

Year	Country	Sex	2.5% Quant.	Median	97.5% Quant.
2013	Belgium	male	4.075209	4.171062	4.310870
2014	Belgium	male	4.082016	4.186349	4.325396
2015	Belgium	male	4.086260	4.202507	4.338838
2016	Belgium	male	4.085840	4.220226	4.354893
2017	Belgium	male	4.084775	4.236993	4.372816
2018	Belgium	male	4.083713	4.255554	4.393480
2019	Belgium	male	4.084788	4.263544	4.412957
2020	Belgium	male	4.085892	4.280969	4.436063
2021	Belgium	male	4.086998	4.297879	4.462347
2022	Belgium	male	4.088104	4.316731	4.490359
2017	Cyprus	male	1.753463	1.918135	2.113676
2018	Cyprus	male	1.824660	1.977191	2.169034
2019	Cyprus	male	1.865503	2.020889	2.230358
2020	Cyprus	male	1.854917	2.060596	2.317932
2021	Cyprus	male	1.844275	2.101663	2.476660
2022	Cyprus	male	1.816015	2.158657	2.627885
2006	Czechia	male	3.756379	3.965727	4.164976
2007	Czechia	male	3.770497	3.959211	4.134229
2008	Czechia	male	3.775132	3.951706	4.113152
2009	Czechia	male	3.781073	3.947550	4.093206
2010	Czechia	male	3.795763	3.935216	4.073388
2011	Czechia	male	3.802267	3.925542	4.058503
2012	Czechia	male	3.808273	3.916208	4.043924
2013	Czechia	male	3.807268	3.904517	4.032663
2014	Czechia	male	3.800561	3.897899	4.026677
2015	Czechia	male	3.796258	3.898441	4.021133
2016	Czechia	male	3.782812	3.895919	4.023218
2017	Czechia	male	3.763977	3.894924	4.021260
2018	Czechia	male	3.741245	3.892757	4.015659
2019	Czechia	male	3.717177	3.883397	4.015749
2020	Czechia	male	3.687792	3.878948	4.016611
2021	Czechia	male	3.661408	3.869957	4.017663
2022	Czechia	male	3.645380	3.862802	4.021453
2004	Switzerland	male	2.285286	2.411575	2.567788
2005	Switzerland	male	2.322030	2.443835	2.585608
2006	Switzerland	male	2.356833	2.475897	2.603658

(Continued on Next Page...)

Table S4: Estimated NHV by country and sex from 2004-2019 ***(continued)***

Year	Country	Sex	2.5% Quant.	Median	97.5% Quant.
2007	Switzerland	male	2.392161	2.508115	2.622516
2008	Switzerland	male	2.428021	2.542401	2.649753
2009	Switzerland	male	2.464422	2.573775	2.679486
2010	Switzerland	male	2.502315	2.608691	2.709556
2011	Switzerland	male	2.542152	2.639242	2.740295
2012	Switzerland	male	2.585467	2.674719	2.771421
2013	Switzerland	male	2.628621	2.706673	2.807531
2014	Switzerland	male	2.667996	2.740811	2.848302
2015	Switzerland	male	2.702294	2.778900	2.886063
2016	Switzerland	male	2.723814	2.815149	2.924014
2017	Switzerland	male	2.752536	2.858882	2.971912
2018	Switzerland	male	2.788323	2.895651	3.020335
2019	Switzerland	male	2.823681	2.930536	3.069547
2020	Switzerland	male	2.859364	2.971176	3.119562
2021	Switzerland	male	2.895475	3.016268	3.170393
2022	Switzerland	male	2.925196	3.052734	3.222053
2004	Germany	male	3.880529	4.083841	4.217659
2005	Germany	male	3.891122	4.078301	4.200999
2006	Germany	male	3.899053	4.070844	4.186233
2007	Germany	male	3.900183	4.062363	4.172630
2008	Germany	male	3.901369	4.052025	4.160039
2009	Germany	male	3.912579	4.041371	4.147488
2010	Germany	male	3.917304	4.030796	4.136068
2011	Germany	male	3.919616	4.024849	4.128607
2012	Germany	male	3.911728	4.014345	4.125191
2013	Germany	male	3.905226	4.005981	4.115022
2014	Germany	male	3.892991	4.000654	4.108248
2015	Germany	male	3.879362	3.998325	4.106003
2016	Germany	male	3.863147	3.998550	4.107122
2017	Germany	male	3.846830	3.991262	4.113633
2018	Germany	male	3.827880	3.983048	4.125389
2019	Germany	male	3.810506	3.976494	4.134874
2020	Germany	male	3.794603	3.972071	4.146742
2021	Germany	male	3.772015	3.964498	4.158903
2022	Germany	male	3.748775	3.960792	4.171105
2004	Denmark	male	2.113838	2.229624	2.344852

(Continued on Next Page...)

Table S4: Estimated NHV by country and sex from 2004-2019 ***(continued)***

Year	Country	Sex	2.5% Quant.	Median	97.5% Quant.
2005	Denmark	male	2.155025	2.261982	2.370705
2006	Denmark	male	2.196922	2.294652	2.400002
2007	Denmark	male	2.234987	2.328285	2.429858
2008	Denmark	male	2.273205	2.360738	2.457329
2009	Denmark	male	2.308850	2.396298	2.486311
2010	Denmark	male	2.339886	2.432487	2.517746
2011	Denmark	male	2.371347	2.468042	2.549649
2012	Denmark	male	2.408790	2.506387	2.582297
2013	Denmark	male	2.451103	2.543085	2.623545
2014	Denmark	male	2.492376	2.581734	2.664210
2015	Denmark	male	2.526747	2.619157	2.709997
2016	Denmark	male	2.559962	2.656521	2.759732
2017	Denmark	male	2.596059	2.696212	2.810565
2018	Denmark	male	2.632531	2.737158	2.861284
2019	Denmark	male	2.669349	2.774810	2.912520
2020	Denmark	male	2.703145	2.815101	2.964678
2021	Denmark	male	2.735676	2.857313	3.017773
2022	Denmark	male	2.768526	2.898941	3.071823
2010	Estonia	male	2.285376	2.407619	2.515785
2011	Estonia	male	2.321620	2.435062	2.531984
2012	Estonia	male	2.354188	2.461547	2.554775
2013	Estonia	male	2.387216	2.485227	2.576618
2014	Estonia	male	2.420712	2.508629	2.607167
2015	Estonia	male	2.451066	2.532520	2.639991
2016	Estonia	male	2.482253	2.560670	2.662277
2017	Estonia	male	2.500171	2.588182	2.691715
2018	Estonia	male	2.512945	2.617084	2.728164
2019	Estonia	male	2.521022	2.649383	2.765736
2020	Estonia	male	2.533602	2.680576	2.809078
2021	Estonia	male	2.546200	2.709136	2.856455
2022	Estonia	male	2.558527	2.742129	2.904632
2004	Spain	male	3.347348	3.498001	3.688898
2005	Spain	male	3.297239	3.432552	3.613407
2006	Spain	male	3.246230	3.369188	3.537009
2007	Spain	male	3.194815	3.311524	3.459134
2008	Spain	male	3.142192	3.259413	3.377703

(Continued on Next Page...)

Table S4: Estimated NHV by country and sex from 2004-2019 ***(continued)***

Year	Country	Sex	2.5% Quant.	Median	97.5% Quant.
2009	Spain	male	3.090435	3.200742	3.300218
2010	Spain	male	3.039625	3.144317	3.231704
2011	Spain	male	2.989466	3.088112	3.171840
2012	Spain	male	2.939894	3.033418	3.117152
2013	Spain	male	2.889127	2.982913	3.061398
2014	Spain	male	2.839237	2.925885	3.008013
2015	Spain	male	2.790210	2.870999	2.951013
2016	Spain	male	2.740510	2.822379	2.903303
2017	Spain	male	2.690462	2.771261	2.858577
2018	Spain	male	2.641328	2.722688	2.813158
2019	Spain	male	2.586018	2.674989	2.769134
2020	Spain	male	2.528921	2.628114	2.727162
2021	Spain	male	2.475114	2.581005	2.686196
2022	Spain	male	2.422000	2.534916	2.645852
2004	France	male	3.954110	4.101470	4.299594
2005	France	male	3.902900	4.035913	4.220847
2006	France	male	3.852355	3.969498	4.143909
2007	France	male	3.799137	3.908429	4.070009
2008	France	male	3.750196	3.854564	4.000267
2009	France	male	3.697364	3.798958	3.929466
2010	France	male	3.645561	3.741282	3.860323
2011	France	male	3.589038	3.685064	3.792560
2012	France	male	3.529655	3.625250	3.725994
2013	France	male	3.469672	3.564048	3.667587
2014	France	male	3.410708	3.510265	3.618347
2015	France	male	3.352746	3.452330	3.568869
2016	France	male	3.295770	3.393085	3.518156
2017	France	male	3.239762	3.342080	3.460407
2018	France	male	3.184701	3.286363	3.407942
2019	France	male	3.130243	3.235844	3.357987
2020	France	male	3.075435	3.182166	3.309031
2021	France	male	3.022239	3.133501	3.263499
2022	France	male	2.968287	3.086178	3.221732
2017	Finland	male	1.776112	1.912624	2.029705
2018	Finland	male	1.828131	1.937079	2.073979
2019	Finland	male	1.838486	1.963458	2.120321

(Continued on Next Page...)

Table S4: Estimated NHV by country and sex from 2004-2019 ***(continued)***

Year	Country	Sex	2.5% Quant.	Median	97.5% Quant.
2020	Finland	male	1.821745	1.989490	2.217819
2021	Finland	male	1.797792	2.022566	2.329718
2022	Finland	male	1.762212	2.061379	2.437676
2004	Greece	male	2.336756	2.455857	2.546893
2005	Greece	male	2.327260	2.437612	2.520586
2006	Greece	male	2.320755	2.420404	2.496679
2007	Greece	male	2.310035	2.401591	2.473771
2008	Greece	male	2.295108	2.383327	2.451333
2009	Greece	male	2.282930	2.364572	2.431897
2010	Greece	male	2.270677	2.345659	2.414553
2011	Greece	male	2.255879	2.325607	2.397333
2012	Greece	male	2.239666	2.306747	2.380236
2013	Greece	male	2.222699	2.288724	2.366024
2014	Greece	male	2.203847	2.269812	2.351651
2015	Greece	male	2.184191	2.255534	2.330872
2016	Greece	male	2.163298	2.237645	2.318205
2017	Greece	male	2.142575	2.221655	2.305785
2018	Greece	male	2.121995	2.202893	2.293656
2019	Greece	male	2.101430	2.183801	2.281607
2020	Greece	male	2.080759	2.166986	2.271962
2021	Greece	male	2.060125	2.148391	2.260097
2022	Greece	male	2.039696	2.129329	2.249710
2015	Croatia	male	2.818899	2.963210	3.125338
2016	Croatia	male	2.931890	3.079602	3.211470
2017	Croatia	male	3.061638	3.202042	3.325886
2018	Croatia	male	3.162173	3.327052	3.474513
2019	Croatia	male	3.266322	3.459496	3.634036
2020	Croatia	male	3.348277	3.588136	3.809929
2021	Croatia	male	3.420714	3.723385	3.987053
2022	Croatia	male	3.504930	3.868867	4.182924
2011	Hungary	male	3.737053	3.961451	4.198961
2012	Hungary	male	3.694354	3.912721	4.111955
2013	Hungary	male	3.655128	3.845653	4.035131
2014	Hungary	male	3.603356	3.786226	3.994130
2015	Hungary	male	3.547816	3.738637	3.937473
2016	Hungary	male	3.493081	3.678122	3.891035

(Continued on Next Page...)

Table S4: Estimated NHV by country and sex from 2004-2019 *(continued)*

Year	Country	Sex	2.5% Quant.	Median	97.5% Quant.
2017	Hungary	male	3.433541	3.628272	3.849194
2018	Hungary	male	3.377104	3.581787	3.819606
2019	Hungary	male	3.310496	3.527093	3.811497
2020	Hungary	male	3.239111	3.476036	3.801276
2021	Hungary	male	3.159718	3.423566	3.781077
2022	Hungary	male	3.082273	3.373404	3.761957
2004	Italy	male	3.638012	3.792002	3.940272
2005	Italy	male	3.655998	3.804094	3.945304
2006	Italy	male	3.677391	3.817871	3.950347
2007	Italy	male	3.701692	3.826553	3.955567
2008	Italy	male	3.721287	3.836572	3.966581
2009	Italy	male	3.742348	3.855299	3.981572
2010	Italy	male	3.755036	3.870574	3.993130
2011	Italy	male	3.762534	3.881574	4.003240
2012	Italy	male	3.774827	3.893342	4.013815
2013	Italy	male	3.785281	3.909547	4.033153
2014	Italy	male	3.793788	3.923897	4.053603
2015	Italy	male	3.802878	3.939104	4.068472
2016	Italy	male	3.814293	3.951878	4.083120
2017	Italy	male	3.819651	3.966996	4.105077
2018	Italy	male	3.820580	3.983181	4.129362
2019	Italy	male	3.821325	3.998240	4.153798
2020	Italy	male	3.822070	4.016486	4.178386
2021	Italy	male	3.822815	4.029089	4.203538
2022	Italy	male	3.823559	4.043919	4.228974
2017	Lithuania	male	1.966791	2.161937	2.320051
2018	Lithuania	male	2.119839	2.294761	2.443565
2019	Lithuania	male	2.257654	2.431049	2.598693
2020	Lithuania	male	2.367347	2.583298	2.808093
2021	Lithuania	male	2.480978	2.745333	3.056385
2022	Lithuania	male	2.573293	2.914560	3.359269
2013	Luxembourg	male	3.629288	3.914485	4.153103
2014	Luxembourg	male	3.750787	4.008530	4.237594
2015	Luxembourg	male	3.863161	4.127877	4.337275
2016	Luxembourg	male	3.978641	4.238819	4.469403
2017	Luxembourg	male	4.095715	4.367421	4.622146

(Continued on Next Page...)

Table S4: Estimated NHV by country and sex from 2004-2019 *(continued)*

Year	Country	Sex	2.5% Quant.	Median	97.5% Quant.
2018	Luxembourg	male	4.199950	4.488704	4.750377
2019	Luxembourg	male	4.303494	4.618392	4.900402
2020	Luxembourg	male	4.399428	4.755745	5.124929
2021	Luxembourg	male	4.480262	4.896041	5.323812
2022	Luxembourg	male	4.571260	5.014299	5.529228
2017	Latvia	male	2.057815	2.223410	2.417324
2018	Latvia	male	2.183627	2.330396	2.513813
2019	Latvia	male	2.256063	2.440169	2.665699
2020	Latvia	male	2.291690	2.559015	2.888447
2021	Latvia	male	2.315060	2.680334	3.102717
2022	Latvia	male	2.339417	2.810347	3.348298
2017	Malta	male	2.069205	2.245738	2.423255
2018	Malta	male	2.053578	2.213989	2.392156
2019	Malta	male	2.010926	2.183875	2.366208
2020	Malta	male	1.932824	2.161301	2.377905
2021	Malta	male	1.869101	2.129763	2.428568
2022	Malta	male	1.795742	2.104780	2.473381
2004	Netherlands	male	2.341247	2.445362	2.574740
2005	Netherlands	male	2.385838	2.488632	2.612018
2006	Netherlands	male	2.430836	2.532519	2.655612
2007	Netherlands	male	2.485407	2.576640	2.694141
2008	Netherlands	male	2.539309	2.624417	2.731684
2009	Netherlands	male	2.589852	2.670062	2.771262
2010	Netherlands	male	2.636201	2.715317	2.813063
2011	Netherlands	male	2.679952	2.761771	2.856186
2012	Netherlands	male	2.720350	2.812735	2.902204
2013	Netherlands	male	2.763426	2.860812	2.950651
2014	Netherlands	male	2.807184	2.913119	3.008147
2015	Netherlands	male	2.851116	2.969082	3.067172
2016	Netherlands	male	2.891873	3.022309	3.129340
2017	Netherlands	male	2.931165	3.073998	3.194306
2018	Netherlands	male	2.971659	3.125741	3.263563
2019	Netherlands	male	3.018083	3.183243	3.335922
2020	Netherlands	male	3.065121	3.238432	3.415960
2021	Netherlands	male	3.112610	3.293271	3.497872
2022	Netherlands	male	3.159560	3.348987	3.570559

(Continued on Next Page...)

Table S4: Estimated NHV by country and sex from 2004-2019 ***(continued)***

Year	Country	Sex	2.5% Quant.	Median	97.5% Quant.
2006	Poland	male	3.177556	3.363993	3.611440
2007	Poland	male	3.185707	3.351467	3.578940
2008	Poland	male	3.189846	3.337737	3.546882
2009	Poland	male	3.193583	3.327522	3.517528
2010	Poland	male	3.200949	3.314843	3.484600
2011	Poland	male	3.207288	3.303818	3.455098
2012	Poland	male	3.207839	3.300330	3.431305
2013	Poland	male	3.203941	3.288313	3.408860
2014	Poland	male	3.199674	3.279612	3.395817
2015	Poland	male	3.189393	3.267826	3.385257
2016	Poland	male	3.172290	3.263330	3.375812
2017	Poland	male	3.153659	3.248354	3.375024
2018	Poland	male	3.131477	3.234993	3.368743
2019	Poland	male	3.105822	3.225807	3.366856
2020	Poland	male	3.080379	3.216849	3.369025
2021	Poland	male	3.054464	3.208562	3.374845
2022	Poland	male	3.025946	3.198320	3.382920
2011	Portugal	male	2.272455	2.401393	2.579618
2012	Portugal	male	2.302620	2.407835	2.569256
2013	Portugal	male	2.307798	2.412541	2.566413
2014	Portugal	male	2.308548	2.429650	2.564225
2015	Portugal	male	2.307666	2.439369	2.576635
2016	Portugal	male	2.302540	2.450754	2.603310
2017	Portugal	male	2.286131	2.460403	2.633796
2018	Portugal	male	2.267427	2.469431	2.664843
2019	Portugal	male	2.245716	2.478152	2.698444
2020	Portugal	male	2.216033	2.490652	2.747678
2021	Portugal	male	2.182451	2.508151	2.805644
2022	Portugal	male	2.156685	2.522416	2.854331
2017	Romania	male	1.865088	1.984371	2.130991
2018	Romania	male	1.989411	2.103079	2.256252
2019	Romania	male	2.068890	2.233734	2.402204
2020	Romania	male	2.141704	2.364406	2.579603
2021	Romania	male	2.213788	2.505229	2.785167
2022	Romania	male	2.303017	2.659305	3.013512
2004	Sweden	male	1.448451	1.523429	1.611074

(Continued on Next Page...)

Table S4: Estimated NHV by country and sex from 2004-2019 ***(continued)***

Year	Country	Sex	2.5% Quant.	Median	97.5% Quant.
2005	Sweden	male	1.498871	1.572926	1.656019
2006	Sweden	male	1.551047	1.623066	1.702220
2007	Sweden	male	1.604913	1.674941	1.748121
2008	Sweden	male	1.660805	1.728399	1.797049
2009	Sweden	male	1.720712	1.783870	1.847507
2010	Sweden	male	1.780412	1.841917	1.899388
2011	Sweden	male	1.840181	1.901901	1.953293
2012	Sweden	male	1.897795	1.962628	2.012525
2013	Sweden	male	1.958306	2.025201	2.078350
2014	Sweden	male	2.016908	2.091179	2.147002
2015	Sweden	male	2.081239	2.158060	2.211285
2016	Sweden	male	2.146044	2.227174	2.280271
2017	Sweden	male	2.212870	2.301468	2.355540
2018	Sweden	male	2.280838	2.375196	2.436340
2019	Sweden	male	2.346240	2.451948	2.518284
2020	Sweden	male	2.412804	2.531435	2.607695
2021	Sweden	male	2.481146	2.615391	2.698335
2022	Sweden	male	2.552238	2.700723	2.793456
2011	Slovenia	male	2.539055	2.662643	2.835017
2012	Slovenia	male	2.574255	2.688518	2.837687
2013	Slovenia	male	2.609459	2.707287	2.847667
2014	Slovenia	male	2.642271	2.735856	2.848931
2015	Slovenia	male	2.672911	2.754136	2.855295
2016	Slovenia	male	2.688128	2.775179	2.879561
2017	Slovenia	male	2.700513	2.790653	2.903197
2018	Slovenia	male	2.709785	2.821380	2.934738
2019	Slovenia	male	2.717089	2.842528	2.981454
2020	Slovenia	male	2.724286	2.866570	3.027800
2021	Slovenia	male	2.727186	2.881719	3.075243
2022	Slovenia	male	2.722732	2.899404	3.123445
2017	Slovakia	male	2.691845	2.876603	3.078890
2018	Slovakia	male	2.995293	3.166089	3.366906
2019	Slovakia	male	3.271768	3.484018	3.741000
2020	Slovakia	male	3.551516	3.842132	4.172829
2021	Slovakia	male	3.829498	4.235402	4.704391
2022	Slovakia	male	4.128872	4.680766	5.314641

(Continued on Next Page...)

Table S4: Estimated NHV by country and sex from 2004-2019 ***(continued)***

Year	Country	Sex	2.5% Quant.	Median	97.5% Quant.
2004	Austria	female	3.735494	3.915671	4.110365
2005	Austria	female	3.784077	3.957955	4.132839
2006	Austria	female	3.833354	4.001247	4.156065
2007	Austria	female	3.883275	4.045384	4.186932
2008	Austria	female	3.933680	4.082222	4.220465
2009	Austria	female	3.988576	4.121603	4.254267
2010	Austria	female	4.040944	4.161906	4.288339
2011	Austria	female	4.087868	4.199689	4.322685
2012	Austria	female	4.132165	4.237815	4.357333
2013	Austria	female	4.184429	4.276999	4.389360
2014	Austria	female	4.234605	4.316763	4.429903
2015	Austria	female	4.275584	4.358605	4.480788
2016	Austria	female	4.310204	4.403287	4.532076
2017	Austria	female	4.345091	4.449014	4.585876
2018	Austria	female	4.376074	4.496176	4.640846
2019	Austria	female	4.407278	4.538810	4.696478
2020	Austria	female	4.435362	4.583822	4.752780
2021	Austria	female	4.463632	4.634616	4.809759
2022	Austria	female	4.499031	4.684298	4.867805
2017	Bulgaria	female	2.955254	3.118614	3.321842
2018	Bulgaria	female	2.994959	3.162940	3.339415
2019	Bulgaria	female	3.033511	3.212301	3.395708
2020	Bulgaria	female	3.045902	3.247493	3.457382
2021	Bulgaria	female	3.071607	3.303859	3.534979
2022	Bulgaria	female	3.064799	3.345219	3.629517
2004	Belgium	female	4.753337	4.928705	5.084123
2005	Belgium	female	4.768259	4.939547	5.088362
2006	Belgium	female	4.784249	4.949718	5.087760
2007	Belgium	female	4.799884	4.960388	5.090092
2008	Belgium	female	4.822057	4.968367	5.097161
2009	Belgium	female	4.844422	4.979019	5.112787
2010	Belgium	female	4.864963	4.989918	5.127725
2011	Belgium	female	4.885593	5.000024	5.138914
2012	Belgium	female	4.905554	5.011070	5.153849
2013	Belgium	female	4.910867	5.024671	5.169136
2014	Belgium	female	4.915651	5.041120	5.186393

(Continued on Next Page...)

Table S4: Estimated NHV by country and sex from 2004-2019 *(continued)*

Year	Country	Sex	2.5% Quant.	Median	97.5% Quant.
2015	Belgium	female	4.925226	5.050290	5.206600
2016	Belgium	female	4.931914	5.063828	5.226887
2017	Belgium	female	4.935126	5.073770	5.247254
2018	Belgium	female	4.938343	5.084815	5.267701
2019	Belgium	female	4.941566	5.101371	5.288228
2020	Belgium	female	4.944794	5.115536	5.308836
2021	Belgium	female	4.949297	5.128701	5.329524
2022	Belgium	female	4.954104	5.137231	5.349836
2017	Cyprus	female	2.446475	2.630950	2.813923
2018	Cyprus	female	2.470842	2.615918	2.770624
2019	Cyprus	female	2.459626	2.605609	2.777563
2020	Cyprus	female	2.399854	2.600710	2.790719
2021	Cyprus	female	2.336283	2.591249	2.799189
2022	Cyprus	female	2.274287	2.598293	2.863892
2006	Czechia	female	4.599881	4.777091	4.913460
2007	Czechia	female	4.574558	4.745225	4.870537
2008	Czechia	female	4.549155	4.712213	4.827989
2009	Czechia	female	4.523896	4.684113	4.787394
2010	Czechia	female	4.495609	4.653271	4.750305
2011	Czechia	female	4.471246	4.619495	4.718923
2012	Czechia	female	4.446398	4.588185	4.683594
2013	Czechia	female	4.421278	4.555365	4.651769
2014	Czechia	female	4.389729	4.524141	4.625032
2015	Czechia	female	4.359839	4.497901	4.597731
2016	Czechia	female	4.323843	4.467589	4.573183
2017	Czechia	female	4.286892	4.436518	4.549060
2018	Czechia	female	4.260820	4.404281	4.527169
2019	Czechia	female	4.234907	4.372992	4.509752
2020	Czechia	female	4.208398	4.342969	4.492409
2021	Czechia	female	4.170297	4.310345	4.475433
2022	Czechia	female	4.130555	4.280354	4.463742
2004	Switzerland	female	2.626681	2.739179	2.879000
2005	Switzerland	female	2.667778	2.771767	2.905889
2006	Switzerland	female	2.708207	2.805741	2.933115
2007	Switzerland	female	2.747922	2.841061	2.960691
2008	Switzerland	female	2.786662	2.873517	2.988528

(Continued on Next Page...)

Table S4: Estimated NHV by country and sex from 2004-2019 *(continued)*

Year	Country	Sex	2.5% Quant.	Median	97.5% Quant.
2009	Switzerland	female	2.827438	2.909365	3.016853
2010	Switzerland	female	2.860361	2.948323	3.048654
2011	Switzerland	female	2.895703	2.986844	3.080790
2012	Switzerland	female	2.933433	3.022768	3.114332
2013	Switzerland	female	2.965850	3.056185	3.147747
2014	Switzerland	female	2.997005	3.094638	3.180756
2015	Switzerland	female	3.026416	3.132675	3.226170
2016	Switzerland	female	3.053507	3.169798	3.278668
2017	Switzerland	female	3.081347	3.204209	3.321897
2018	Switzerland	female	3.110112	3.244129	3.375464
2019	Switzerland	female	3.139148	3.279264	3.431675
2020	Switzerland	female	3.168340	3.317385	3.491507
2021	Switzerland	female	3.195890	3.360054	3.552381
2022	Switzerland	female	3.224190	3.400590	3.614317
2004	Germany	female	4.608425	4.754839	4.916966
2005	Germany	female	4.599849	4.735299	4.886126
2006	Germany	female	4.586269	4.717227	4.857341
2007	Germany	female	4.572083	4.697857	4.833489
2008	Germany	female	4.557211	4.680821	4.809757
2009	Germany	female	4.545744	4.658635	4.783771
2010	Germany	female	4.530738	4.634310	4.758261
2011	Germany	female	4.513707	4.613086	4.734183
2012	Germany	female	4.490801	4.592418	4.712471
2013	Germany	female	4.467426	4.576897	4.693168
2014	Germany	female	4.444380	4.558130	4.676685
2015	Germany	female	4.422837	4.541738	4.668512
2016	Germany	female	4.404281	4.523478	4.654200
2017	Germany	female	4.382764	4.508930	4.638314
2018	Germany	female	4.358576	4.492731	4.629526
2019	Germany	female	4.327364	4.474504	4.616664
2020	Germany	female	4.295916	4.455082	4.603838
2021	Germany	female	4.264699	4.435746	4.591048
2022	Germany	female	4.237906	4.418943	4.578294
2004	Denmark	female	2.488565	2.605938	2.717544
2005	Denmark	female	2.526139	2.640767	2.740164
2006	Denmark	female	2.564281	2.671154	2.762975

(Continued on Next Page...)

Table S4: Estimated NHV by country and sex from 2004-2019 *(continued)*

Year	Country	Sex	2.5% Quant.	Median	97.5% Quant.
2007	Denmark	female	2.602998	2.704470	2.788135
2008	Denmark	female	2.642300	2.738037	2.818199
2009	Denmark	female	2.680643	2.774369	2.851143
2010	Denmark	female	2.720850	2.809468	2.882828
2011	Denmark	female	2.759447	2.845588	2.925323
2012	Denmark	female	2.801874	2.883952	2.965726
2013	Denmark	female	2.843085	2.918636	3.006261
2014	Denmark	female	2.881737	2.958353	3.044103
2015	Denmark	female	2.913538	2.991723	3.088167
2016	Denmark	female	2.945451	3.032236	3.134852
2017	Denmark	female	2.976872	3.072948	3.185511
2018	Denmark	female	3.009854	3.114324	3.237803
2019	Denmark	female	3.039793	3.149028	3.288694
2020	Denmark	female	3.064266	3.191208	3.337181
2021	Denmark	female	3.091046	3.231469	3.388680
2022	Denmark	female	3.121496	3.271005	3.441476
2010	Estonia	female	2.712585	2.807739	2.885302
2011	Estonia	female	2.740018	2.828649	2.903997
2012	Estonia	female	2.764124	2.849791	2.924152
2013	Estonia	female	2.783987	2.870728	2.943813
2014	Estonia	female	2.803679	2.893038	2.960213
2015	Estonia	female	2.829431	2.911736	2.977041
2016	Estonia	female	2.855434	2.934417	3.003339
2017	Estonia	female	2.872642	2.955193	3.026714
2018	Estonia	female	2.889775	2.977300	3.054309
2019	Estonia	female	2.911482	2.999718	3.082475
2020	Estonia	female	2.928030	3.022951	3.110941
2021	Estonia	female	2.941342	3.045296	3.139833
2022	Estonia	female	2.956208	3.069997	3.168887
2004	Spain	female	4.504645	4.679303	4.882818
2005	Spain	female	4.398208	4.548768	4.739438
2006	Spain	female	4.289840	4.423814	4.600748
2007	Spain	female	4.177624	4.299755	4.466166
2008	Spain	female	4.066658	4.180841	4.335046
2009	Spain	female	3.965286	4.062709	4.206594
2010	Spain	female	3.858072	3.952412	4.078485

(Continued on Next Page...)

Table S4: Estimated NHV by country and sex from 2004-2019 ***(continued)***

Year	Country	Sex	2.5% Quant.	Median	97.5% Quant.
2011	Spain	female	3.748886	3.842315	3.958075
2012	Spain	female	3.642618	3.737187	3.843626
2013	Spain	female	3.545995	3.636059	3.730048
2014	Spain	female	3.441441	3.536817	3.621179
2015	Spain	female	3.344977	3.440848	3.516975
2016	Spain	female	3.251491	3.346616	3.420922
2017	Spain	female	3.156576	3.252368	3.328890
2018	Spain	female	3.063828	3.159841	3.241222
2019	Spain	female	2.975365	3.069551	3.157538
2020	Spain	female	2.889302	2.985033	3.076954
2021	Spain	female	2.805730	2.903955	2.998559
2022	Spain	female	2.723722	2.823784	2.922291
2004	France	female	4.860738	5.026449	5.154656
2005	France	female	4.780526	4.935088	5.053465
2006	France	female	4.703313	4.843147	4.954260
2007	France	female	4.621476	4.749313	4.854717
2008	France	female	4.534260	4.658169	4.757012
2009	France	female	4.443631	4.571115	4.668687
2010	France	female	4.352821	4.481255	4.579245
2011	France	female	4.263869	4.393754	4.492465
2012	France	female	4.185106	4.315402	4.412884
2013	France	female	4.108121	4.233075	4.337252
2014	France	female	4.032322	4.155058	4.256404
2015	France	female	3.953072	4.080264	4.177067
2016	France	female	3.874256	4.005954	4.104518
2017	France	female	3.797011	3.929962	4.034855
2018	France	female	3.721306	3.855256	3.968500
2019	France	female	3.647111	3.783043	3.904226
2020	France	female	3.574809	3.709246	3.840994
2021	France	female	3.502502	3.642894	3.778785
2022	France	female	3.431820	3.574585	3.717584
2017	Finland	female	2.031178	2.149249	2.250289
2018	Finland	female	2.058736	2.175331	2.270138
2019	Finland	female	2.073179	2.197434	2.338795
2020	Finland	female	2.062621	2.218542	2.405112
2021	Finland	female	2.045617	2.246777	2.472311

(Continued on Next Page...)

Table S4: Estimated NHV by country and sex from 2004-2019 ***(continued)***

Year	Country	Sex	2.5% Quant.	Median	97.5% Quant.
2022	Finland	female	2.023181	2.272947	2.547662
2004	Greece	female	3.086101	3.226197	3.356151
2005	Greece	female	3.066892	3.196781	3.325205
2006	Greece	female	3.050109	3.169539	3.294889
2007	Greece	female	3.033393	3.142664	3.258280
2008	Greece	female	3.016713	3.115604	3.222211
2009	Greece	female	2.995061	3.091100	3.190692
2010	Greece	female	2.971392	3.066188	3.163412
2011	Greece	female	2.944319	3.041964	3.136675
2012	Greece	female	2.917457	3.014951	3.110175
2013	Greece	female	2.890841	2.988486	3.080325
2014	Greece	female	2.864467	2.960698	3.054306
2015	Greece	female	2.838334	2.930976	3.028508
2016	Greece	female	2.812440	2.905751	3.003714
2017	Greece	female	2.786782	2.879803	2.982835
2018	Greece	female	2.760716	2.854590	2.957939
2019	Greece	female	2.733438	2.829992	2.933250
2020	Greece	female	2.706431	2.810920	2.908768
2021	Greece	female	2.680167	2.785614	2.885156
2022	Greece	female	2.655134	2.763126	2.866030
2015	Croatia	female	3.662954	3.855509	4.056894
2016	Croatia	female	3.780372	3.953263	4.136959
2017	Croatia	female	3.904693	4.054573	4.226656
2018	Croatia	female	3.990915	4.163482	4.357730
2019	Croatia	female	4.079011	4.268547	4.511060
2020	Croatia	female	4.163259	4.399341	4.657319
2021	Croatia	female	4.231980	4.530046	4.826874
2022	Croatia	female	4.283629	4.642097	5.021880
2011	Hungary	female	4.349776	4.571719	4.764499
2012	Hungary	female	4.309988	4.513142	4.683895
2013	Hungary	female	4.271592	4.460993	4.614039
2014	Hungary	female	4.235215	4.411618	4.555548
2015	Hungary	female	4.199156	4.349337	4.494475
2016	Hungary	female	4.129226	4.305021	4.449953
2017	Hungary	female	4.052095	4.249679	4.403086
2018	Hungary	female	3.971077	4.187805	4.364077

(Continued on Next Page...)

Table S4: Estimated NHV by country and sex from 2004-2019 *(continued)*

Year	Country	Sex	2.5% Quant.	Median	97.5% Quant.
2019	Hungary	female	3.892660	4.132993	4.335398
2020	Hungary	female	3.818134	4.084492	4.311446
2021	Hungary	female	3.745035	4.039111	4.287633
2022	Hungary	female	3.673336	3.987933	4.263960
2004	Italy	female	5.003464	5.146865	5.354525
2005	Italy	female	4.969202	5.107559	5.301872
2006	Italy	female	4.935175	5.069477	5.249347
2007	Italy	female	4.901381	5.030455	5.192950
2008	Italy	female	4.867818	4.991574	5.137161
2009	Italy	female	4.831634	4.953935	5.083678
2010	Italy	female	4.793832	4.915164	5.038097
2011	Italy	female	4.756199	4.876081	4.995010
2012	Italy	female	4.717633	4.837252	4.952291
2013	Italy	female	4.678586	4.802915	4.909938
2014	Italy	female	4.639624	4.767496	4.867947
2015	Italy	female	4.595683	4.729644	4.826315
2016	Italy	female	4.555452	4.692541	4.789670
2017	Italy	female	4.515718	4.654662	4.765324
2018	Italy	female	4.476575	4.618528	4.740883
2019	Italy	female	4.436312	4.583231	4.714252
2020	Italy	female	4.395896	4.548069	4.687772
2021	Italy	female	4.353507	4.512817	4.661439
2022	Italy	female	4.311373	4.474869	4.635255
2017	Lithuania	female	2.567954	2.702133	2.874736
2018	Lithuania	female	2.711482	2.852865	3.012051
2019	Lithuania	female	2.841251	3.007971	3.182122
2020	Lithuania	female	2.969932	3.174886	3.403856
2021	Lithuania	female	3.099896	3.347179	3.643614
2022	Lithuania	female	3.199185	3.501790	3.914059
2013	Luxembourg	female	4.726477	4.949570	5.267130
2014	Luxembourg	female	4.814400	5.058309	5.355194
2015	Luxembourg	female	4.919486	5.158378	5.426783
2016	Luxembourg	female	5.007898	5.263633	5.548727
2017	Luxembourg	female	5.090465	5.368788	5.646570
2018	Luxembourg	female	5.158050	5.484543	5.805448
2019	Luxembourg	female	5.202798	5.601645	5.967219

(Continued on Next Page...)

Table S4: Estimated NHV by country and sex from 2004-2019 ***(continued)***

Year	Country	Sex	2.5% Quant.	Median	97.5% Quant.
2020	Luxembourg	female	5.259403	5.713808	6.102131
2021	Luxembourg	female	5.316630	5.831470	6.244278
2022	Luxembourg	female	5.374484	5.957734	6.409344
2017	Latvia	female	2.535846	2.677892	2.811642
2018	Latvia	female	2.640800	2.792581	2.938614
2019	Latvia	female	2.749407	2.914481	3.056635
2020	Latvia	female	2.834098	3.028720	3.222079
2021	Latvia	female	2.913740	3.168969	3.415335
2022	Latvia	female	2.997064	3.310926	3.632609
2017	Malta	female	2.377570	2.555494	2.711631
2018	Malta	female	2.376073	2.539929	2.686867
2019	Malta	female	2.347676	2.524545	2.679804
2020	Malta	female	2.311834	2.513243	2.682229
2021	Malta	female	2.256573	2.493475	2.700241
2022	Malta	female	2.196017	2.479374	2.728162
2004	Netherlands	female	2.602690	2.705632	2.824526
2005	Netherlands	female	2.659001	2.759838	2.874629
2006	Netherlands	female	2.717502	2.819180	2.922157
2007	Netherlands	female	2.777291	2.874020	2.973891
2008	Netherlands	female	2.838394	2.933740	3.038758
2009	Netherlands	female	2.902079	2.988639	3.098946
2010	Netherlands	female	2.965532	3.049264	3.159039
2011	Netherlands	female	3.030461	3.110214	3.218458
2012	Netherlands	female	3.095581	3.176008	3.282548
2013	Netherlands	female	3.157581	3.241965	3.348358
2014	Netherlands	female	3.218416	3.311928	3.424295
2015	Netherlands	female	3.279749	3.379642	3.497853
2016	Netherlands	female	3.342252	3.444922	3.573988
2017	Netherlands	female	3.406840	3.514502	3.653512
2018	Netherlands	female	3.473827	3.588371	3.738175
2019	Netherlands	female	3.538273	3.659044	3.825366
2020	Netherlands	female	3.604024	3.733569	3.923212
2021	Netherlands	female	3.671078	3.807553	4.023717
2022	Netherlands	female	3.739123	3.881499	4.126797
2006	Poland	female	4.032279	4.232842	4.537913
2007	Poland	female	4.022716	4.209174	4.488761

(Continued on Next Page...)

Table S4: Estimated NHV by country and sex from 2004-2019 *(continued)*

Year	Country	Sex	2.5% Quant.	Median	97.5% Quant.
2008	Poland	female	4.013176	4.187799	4.442965
2009	Poland	female	4.001438	4.165724	4.397641
2010	Poland	female	3.986298	4.149503	4.352784
2011	Poland	female	3.979180	4.126305	4.307338
2012	Poland	female	3.974777	4.109124	4.264534
2013	Poland	female	3.963227	4.087104	4.235236
2014	Poland	female	3.950580	4.067836	4.205268
2015	Poland	female	3.937463	4.046506	4.170202
2016	Poland	female	3.918957	4.031999	4.138782
2017	Poland	female	3.894375	4.006397	4.120388
2018	Poland	female	3.866016	3.986087	4.105609
2019	Poland	female	3.834256	3.967192	4.084215
2020	Poland	female	3.802758	3.942779	4.074831
2021	Poland	female	3.769017	3.921824	4.067606
2022	Poland	female	3.736439	3.899084	4.062755
2011	Portugal	female	2.853600	2.993550	3.171013
2012	Portugal	female	2.848492	2.970889	3.143723
2013	Portugal	female	2.823703	2.937229	3.115895
2014	Portugal	female	2.791781	2.911360	3.076875
2015	Portugal	female	2.750132	2.896809	3.043829
2016	Portugal	female	2.714950	2.872076	3.026457
2017	Portugal	female	2.680219	2.846310	3.021533
2018	Portugal	female	2.632801	2.817884	3.028163
2019	Portugal	female	2.577436	2.794656	3.016033
2020	Portugal	female	2.523136	2.768821	3.003956
2021	Portugal	female	2.469982	2.746178	2.991932
2022	Portugal	female	2.418891	2.720391	2.979961
2017	Romania	female	2.486490	2.638569	2.798828
2018	Romania	female	2.631490	2.760667	2.921779
2019	Romania	female	2.763753	2.890966	3.056924
2020	Romania	female	2.884204	3.023422	3.190795
2021	Romania	female	2.981762	3.160975	3.411926
2022	Romania	female	3.078163	3.311118	3.627887
2004	Sweden	female	1.743347	1.822340	1.890626
2005	Sweden	female	1.790677	1.864647	1.931379
2006	Sweden	female	1.839293	1.907955	1.973010

(Continued on Next Page...)

Table S4: Estimated NHV by country and sex from 2004-2019 *(continued)*

Year	Country	Sex	2.5% Quant.	Median	97.5% Quant.
2007	Sweden	female	1.889229	1.953785	2.015539
2008	Sweden	female	1.940252	2.002405	2.060917
2009	Sweden	female	1.991951	2.053001	2.110539
2010	Sweden	female	2.045027	2.105433	2.159884
2011	Sweden	female	2.099472	2.158224	2.213027
2012	Sweden	female	2.153431	2.210306	2.268493
2013	Sweden	female	2.206032	2.266084	2.325078
2014	Sweden	female	2.260673	2.321541	2.383097
2015	Sweden	female	2.318576	2.378664	2.442661
2016	Sweden	female	2.372628	2.439669	2.508474
2017	Sweden	female	2.427096	2.497659	2.576503
2018	Sweden	female	2.482761	2.556905	2.646281
2019	Sweden	female	2.539709	2.618921	2.721636
2020	Sweden	female	2.594627	2.680676	2.799340
2021	Sweden	female	2.650392	2.744825	2.879263
2022	Sweden	female	2.707356	2.817257	2.961468
2011	Slovenia	female	2.638634	2.766283	2.851495
2012	Slovenia	female	2.676077	2.786193	2.865845
2013	Slovenia	female	2.711739	2.811347	2.880106
2014	Slovenia	female	2.740556	2.839933	2.905399
2015	Slovenia	female	2.776596	2.862361	2.934632
2016	Slovenia	female	2.802885	2.889001	2.963710
2017	Slovenia	female	2.838022	2.917714	2.996552
2018	Slovenia	female	2.864151	2.940017	3.035647
2019	Slovenia	female	2.883440	2.962796	3.078287
2020	Slovenia	female	2.899387	2.993771	3.115848
2021	Slovenia	female	2.908510	3.025173	3.156026
2022	Slovenia	female	2.917295	3.057086	3.199539
2017	Slovakia	female	3.352496	3.547030	3.760956
2018	Slovakia	female	3.545181	3.731166	3.944140
2019	Slovakia	female	3.698529	3.921240	4.131305
2020	Slovakia	female	3.838485	4.129527	4.428900
2021	Slovakia	female	3.984441	4.345077	4.734480
2022	Slovakia	female	4.135798	4.589135	5.085681

Table S5: Estimated NHV by epidemic period

Country	Sex	Period	2.5% Quant.	Median	97.5% Quant.
Austria	male	Epidemic	0.2961026	0.3247069	0.3652698
Bulgaria	male	Epidemic	0.1389198	0.1589565	0.1893942
Belgium	male	Epidemic	0.2676780	0.2938237	0.3168746
Cyprus	male	Epidemic	0.1381502	0.1759684	0.2235481
Czechia	male	Epidemic	0.1863108	0.2094735	0.2302330
Switzerland	male	Epidemic	0.2514801	0.2815730	0.3118547
Germany	male	Epidemic	0.4073919	0.4527449	0.4930440
Denmark	male	Epidemic	0.2845631	0.3158190	0.3570682
Estonia	male	Epidemic	0.0802252	0.0878454	0.0978270
Spain	male	Epidemic	0.1903019	0.2093903	0.2421676
France	male	Epidemic	0.4052236	0.4399576	0.4828990
Finland	male	Epidemic	0.3531941	0.4059161	0.4850489
Greece	male	Epidemic	0.1338503	0.1444834	0.1596465
Croatia	male	Epidemic	0.0618355	0.0713298	0.0836418
Hungary	male	Epidemic	0.0836844	0.0982155	0.1232349
Italy	male	Epidemic	0.0583614	0.0631818	0.0702774
Lithuania	male	Epidemic	0.0930986	0.1072762	0.1250641
Luxembourg	male	Epidemic	0.1078166	0.1251759	0.1456070
Latvia	male	Epidemic	0.1277719	0.1528351	0.1765361
Malta	male	Epidemic	0.1543045	0.1852309	0.2188238
Netherlands	male	Epidemic	0.1516117	0.1737363	0.2078641
Poland	male	Epidemic	0.1424516	0.1566970	0.1722637
Portugal	male	Epidemic	0.1830262	0.2182611	0.2640315
Romania	male	Epidemic	0.1614400	0.1928857	0.2182646
Sweden	male	Epidemic	0.3168408	0.3538661	0.3993035
Slovenia	male	Epidemic	0.1150790	0.1311057	0.1460913
Slovakia	male	Epidemic	0.0930273	0.1086417	0.1255407
Austria	male	Post-Epidemic	0.8530829	0.9099770	0.9743933
Bulgaria	male	Post-Epidemic	0.7823400	0.9177289	1.0831375
Belgium	male	Post-Epidemic	0.9876400	1.0568858	1.1170280
Cyprus	male	Post-Epidemic	1.1772485	1.3574490	1.5908075
Czechia	male	Post-Epidemic	1.0526479	1.1321710	1.2019796
Switzerland	male	Post-Epidemic	0.9279105	1.0115468	1.0885385
Germany	male	Post-Epidemic	1.0606909	1.1125397	1.1676724
Denmark	male	Post-Epidemic	0.8544293	0.9177864	0.9828771
Estonia	male	Post-Epidemic	1.0618199	1.1277465	1.2060713

(Continued on Next Page...)

Table S5: Estimated NHV by epidemic period *(continued)*

Country	Sex	Period	2.5% Quant.	Median	97.5% Quant.
Spain	male	Post-Epidemic	0.9280577	1.0082207	1.0901918
France	male	Post-Epidemic	1.0617575	1.1214084	1.1929081
Finland	male	Post-Epidemic	0.8313261	0.9560583	1.1258789
Greece	male	Post-Epidemic	0.9942394	1.0576204	1.1224754
Croatia	male	Post-Epidemic	0.8458377	0.9362992	1.0273226
Hungary	male	Post-Epidemic	0.7822745	0.8747332	0.9494905
Italy	male	Post-Epidemic	0.8603274	0.9059928	0.9544611
Lithuania	male	Post-Epidemic	0.8358249	0.9385477	1.0637289
Luxembourg	male	Post-Epidemic	0.7373418	0.8069946	0.8905483
Latvia	male	Post-Epidemic	0.7347193	0.8916349	1.0361230
Malta	male	Post-Epidemic	0.7677525	0.8893956	1.0293716
Netherlands	male	Post-Epidemic	0.8447200	0.9104174	0.9732034
Poland	male	Post-Epidemic	0.7950836	0.8511499	0.9138764
Portugal	male	Post-Epidemic	0.8181195	0.9463074	1.0935674
Romania	male	Post-Epidemic	0.7791793	0.8719217	1.0127820
Sweden	male	Post-Epidemic	0.9060374	0.9660835	1.0278839
Slovenia	male	Post-Epidemic	0.8254595	0.9041156	0.9735542
Slovakia	male	Post-Epidemic	0.5620056	0.6406490	0.7110576
Austria	female	Epidemic	0.2837441	0.3068262	0.3317178
Bulgaria	female	Epidemic	0.1291488	0.1500241	0.1718772
Belgium	female	Epidemic	0.2207433	0.2340601	0.2484315
Cyprus	female	Epidemic	0.0950042	0.1135314	0.1340445
Czechia	female	Epidemic	0.1671847	0.1791001	0.1917894
Switzerland	female	Epidemic	0.2034044	0.2244011	0.2410499
Germany	female	Epidemic	0.3927454	0.4196070	0.4479282
Denmark	female	Epidemic	0.2805334	0.3055506	0.3300202
Estonia	female	Epidemic	0.0712495	0.0782305	0.0842722
Spain	female	Epidemic	0.1682595	0.1819127	0.2007120
France	female	Epidemic	0.3525840	0.3874634	0.4147254
Finland	female	Epidemic	0.3581683	0.4055745	0.4508862
Greece	female	Epidemic	0.1057749	0.1136848	0.1224595
Croatia	female	Epidemic	0.0578211	0.0651453	0.0749464
Hungary	female	Epidemic	0.0766009	0.0888672	0.1025201
Italy	female	Epidemic	0.0470562	0.0511942	0.0551493
Lithuania	female	Epidemic	0.0929536	0.1048487	0.1191917
Luxembourg	female	Epidemic	0.0964758	0.1087169	0.1261391

(Continued on Next Page...)

Table S5: Estimated NHV by epidemic period *(continued)*

Country	Sex	Period	2.5% Quant.	Median	97.5% Quant.
Latvia	female	Epidemic	0.1065353	0.1249193	0.1418781
Malta	female	Epidemic	0.1479663	0.1649290	0.1891140
Netherlands	female	Epidemic	0.1403771	0.1586452	0.1900386
Poland	female	Epidemic	0.1270995	0.1359868	0.1483919
Portugal	female	Epidemic	0.1736436	0.1994242	0.2315088
Romania	female	Epidemic	0.1728846	0.1927035	0.2154959
Sweden	female	Epidemic	0.2800377	0.3115416	0.3483092
Slovenia	female	Epidemic	0.1117062	0.1205907	0.1305004
Slovakia	female	Epidemic	0.0989316	0.1143543	0.1343923
Austria	female	Post-Epidemic	0.8970601	0.9537846	0.9995732
Bulgaria	female	Post-Epidemic	0.8831858	0.9816891	1.0838686
Belgium	female	Post-Epidemic	1.0050206	1.0457638	1.0793023
Cyprus	female	Post-Epidemic	1.0161588	1.1447752	1.2968389
Czechia	female	Post-Epidemic	1.0380854	1.0911240	1.1511960
Switzerland	female	Post-Epidemic	0.9392716	0.9991395	1.0668715
Germany	female	Post-Epidemic	1.0386183	1.0915676	1.1482201
Denmark	female	Post-Epidemic	0.8576652	0.9226262	0.9829440
Estonia	female	Post-Epidemic	1.0423757	1.0938975	1.1456008
Spain	female	Post-Epidemic	0.9399712	1.0020520	1.0657079
France	female	Post-Epidemic	1.0603268	1.1067383	1.1602217
Finland	female	Post-Epidemic	0.8455450	0.9383145	1.0711127
Greece	female	Post-Epidemic	0.9760275	1.0199925	1.0700552
Croatia	female	Post-Epidemic	0.8539702	0.9280852	1.0061937
Hungary	female	Post-Epidemic	0.8440750	0.9161328	0.9977147
Italy	female	Post-Epidemic	0.8682464	0.9125841	0.9631327
Lithuania	female	Post-Epidemic	0.9462452	1.0370617	1.1307461
Luxembourg	female	Post-Epidemic	0.7767598	0.8491808	0.9368589
Latvia	female	Post-Epidemic	0.8257720	0.9113118	1.0012395
Malta	female	Post-Epidemic	0.8062984	0.8945664	1.0215910
Netherlands	female	Post-Epidemic	0.8102648	0.8620882	0.9175611
Poland	female	Post-Epidemic	0.7668290	0.8040323	0.8430228
Portugal	female	Post-Epidemic	0.8756009	0.9820132	1.0843554
Romania	female	Post-Epidemic	0.8100965	0.8870977	0.9617592
Sweden	female	Post-Epidemic	0.9243669	0.9827051	1.0419995
Slovenia	female	Post-Epidemic	0.8927724	0.9400193	0.9970225
Slovakia	female	Post-Epidemic	0.6406450	0.7149784	0.7858796

Table S6: Estimated relative change by age and epidemic period

Age	Sex	Period	2.5% Quant.	Median	97.5% Quant.
50	male	Pre-	0.6222053	0.6533698	0.6833842
51	male	Pre-	0.6416818	0.6656251	0.6879020
52	male	Pre-	0.6592677	0.6815937	0.6994560
53	male	Pre-	0.6776096	0.6970969	0.7142328
54	male	Pre-	0.6924641	0.7139882	0.7333720
55	male	Pre-	0.7089068	0.7311390	0.7527415
56	male	Pre-	0.7289271	0.7495422	0.7696026
57	male	Pre-	0.7505231	0.7690813	0.7868127
58	male	Pre-	0.7662270	0.7854083	0.8074882
59	male	Pre-	0.7804060	0.8007088	0.8222629
60	male	Pre-	0.7956348	0.8142463	0.8348948
61	male	Pre-	0.8042011	0.8257226	0.8456175
62	male	Pre-	0.8157176	0.8369881	0.8554958
63	male	Pre-	0.8301705	0.8472905	0.8687556
64	male	Pre-	0.8416070	0.8598079	0.8795001
65	male	Pre-	0.8542529	0.8732766	0.8938377
66	male	Pre-	0.8677460	0.8894433	0.9065529
67	male	Pre-	0.8849658	0.9048353	0.9252388
68	male	Pre-	0.9047495	0.9224522	0.9430781
69	male	Pre-	0.9158714	0.9405733	0.9618433
70	male	Pre-	0.9318820	0.9566880	0.9795191
71	male	Pre-	0.9485974	0.9737198	0.9954537
72	male	Pre-	0.9658392	0.9894204	1.0105642
73	male	Pre-	0.9835678	1.0058701	1.0296385
74	male	Pre-	0.9970748	1.0248482	1.0449809
75	male	Pre-	1.0137271	1.0409441	1.0617639
76	male	Pre-	1.0308115	1.0581002	1.0811283
77	male	Pre-	1.0453467	1.0746535	1.0995508
78	male	Pre-	1.0629160	1.0924531	1.1202138
79	male	Pre-	1.0785049	1.1101039	1.1348833
80	male	Pre-	1.0944649	1.1267834	1.1555908
81	male	Pre-	1.1049074	1.1420933	1.1727871
82	male	Pre-	1.1198229	1.1594414	1.1901931
83	male	Pre-	1.1361074	1.1732528	1.2098521
84	male	Pre-	1.1477130	1.1849660	1.2216739
85	male	Pre-	1.1579440	1.1976237	1.2324224

(Continued on Next Page...)

Table S6: Estimated relative change by age and epidemic period *(continued)*

Age	Sex	Period	2.5% Quant.	Median	97.5% Quant.
86	male	Pre-	1.1734826	1.2093617	1.2386285
87	male	Pre-	1.1816384	1.2175757	1.2472776
88	male	Pre-	1.1866461	1.2235984	1.2584300
89	male	Pre-	1.1899392	1.2303481	1.2616889
90	male	Pre-	1.1946845	1.2375402	1.2724510
91	male	Pre-	1.2013122	1.2416181	1.2810022
92	male	Pre-	1.2100312	1.2459908	1.2920233
93	male	Pre-	1.2128703	1.2542417	1.3035071
94	male	Pre-	1.2133847	1.2625152	1.3279619
95	male	Pre-	1.2099136	1.2688088	1.3506962
96	male	Pre-	1.1999127	1.2703347	1.3713414
97	male	Pre-	1.1931920	1.2741572	1.3981606
98	male	Pre-	1.1761062	1.2811552	1.4326137
99	male	Pre-	1.1560347	1.2911998	1.4580449
100	male	Pre-	1.1344307	1.3053740	1.4773485
50	male	Epi-	0.6883154	0.8104052	0.9898002
51	male	Epi-	0.7142334	0.8246640	0.9841284
52	male	Epi-	0.7357728	0.8389158	0.9748656
53	male	Epi-	0.7622906	0.8501604	0.9591878
54	male	Epi-	0.7896488	0.8654134	0.9486859
55	male	Epi-	0.8145691	0.8843014	0.9509256
56	male	Epi-	0.8380623	0.9020646	0.9620791
57	male	Epi-	0.8674671	0.9172548	0.9660522
58	male	Epi-	0.8892424	0.9325655	0.9830726
59	male	Epi-	0.9065305	0.9525965	1.0009867
60	male	Epi-	0.9242783	0.9694613	1.0160338
61	male	Epi-	0.9346854	0.9875483	1.0322758
62	male	Epi-	0.9463376	1.0051316	1.0510993
63	male	Epi-	0.9585794	1.0207241	1.0704292
64	male	Epi-	0.9753638	1.0369576	1.0930384
65	male	Epi-	0.9924421	1.0517322	1.1075837
66	male	Epi-	1.0116853	1.0665473	1.1312740
67	male	Epi-	1.0229519	1.0810994	1.1497987
68	male	Epi-	1.0336813	1.0919364	1.1619851
69	male	Epi-	1.0463344	1.1022848	1.1646383
70	male	Epi-	1.0569905	1.1099301	1.1764088

(Continued on Next Page...)

Table S6: Estimated relative change by age and epidemic period *(continued)*

Age	Sex	Period	2.5% Quant.	Median	97.5% Quant.
71	male	Epi-	1.0654733	1.1171033	1.1892558
72	male	Epi-	1.0717503	1.1248830	1.1998850
73	male	Epi-	1.0702287	1.1314607	1.2032970
74	male	Epi-	1.0762820	1.1352214	1.2055469
75	male	Epi-	1.0869140	1.1417025	1.2181176
76	male	Epi-	1.0951474	1.1472433	1.2177877
77	male	Epi-	1.0992321	1.1513562	1.2281963
78	male	Epi-	1.0971388	1.1518003	1.2294282
79	male	Epi-	1.0918210	1.1510440	1.2314422
80	male	Epi-	1.0884366	1.1517090	1.2267811
81	male	Epi-	1.0833947	1.1439691	1.2191653
82	male	Epi-	1.0752461	1.1365337	1.2194371
83	male	Epi-	1.0640121	1.1268748	1.2063660
84	male	Epi-	1.0550471	1.1144722	1.1827760
85	male	Epi-	1.0377403	1.0991675	1.1618227
86	male	Epi-	1.0266644	1.0825446	1.1346798
87	male	Epi-	1.0119757	1.0635615	1.1119161
88	male	Epi-	0.9989992	1.0495251	1.0996934
89	male	Epi-	0.9796118	1.0283914	1.0818339
90	male	Epi-	0.9489894	1.0066059	1.0648148
91	male	Epi-	0.9225675	0.9834939	1.0422294
92	male	Epi-	0.8895485	0.9597697	1.0192369
93	male	Epi-	0.8545814	0.9336864	1.0079216
94	male	Epi-	0.8211315	0.9108288	1.0021571
95	male	Epi-	0.7910191	0.8905596	0.9956785
96	male	Epi-	0.7566473	0.8662451	0.9879954
97	male	Epi-	0.7281744	0.8513442	0.9848695
98	male	Epi-	0.6910998	0.8299928	0.9813572
99	male	Epi-	0.6523472	0.8123359	0.9822552
100	male	Epi-	0.6160206	0.7910277	0.9797077
50	male	Post-	0.6707334	0.7630509	0.8367007
51	male	Post-	0.6926570	0.7641664	0.8256209
52	male	Post-	0.7057793	0.7695621	0.8164827
53	male	Post-	0.7246961	0.7726911	0.8187910
54	male	Post-	0.7400303	0.7794174	0.8226015
55	male	Post-	0.7527089	0.7877303	0.8212115

(Continued on Next Page...)

Table S6: Estimated relative change by age and epidemic period *(continued)*

Age	Sex	Period	2.5% Quant.	Median	97.5% Quant.
56	male	Post-	0.7694026	0.7966090	0.8244880
57	male	Post-	0.7762740	0.8064949	0.8333265
58	male	Post-	0.7841614	0.8159495	0.8442348
59	male	Post-	0.7931043	0.8252855	0.8546448
60	male	Post-	0.8041475	0.8341772	0.8653198
61	male	Post-	0.8102126	0.8433129	0.8752219
62	male	Post-	0.8171678	0.8530909	0.8846452
63	male	Post-	0.8276646	0.8613219	0.8936457
64	male	Post-	0.8373883	0.8705070	0.8988273
65	male	Post-	0.8499301	0.8793692	0.9089531
66	male	Post-	0.8622299	0.8898914	0.9213583
67	male	Post-	0.8787459	0.9044013	0.9364049
68	male	Post-	0.8931209	0.9201839	0.9511252
69	male	Post-	0.9089324	0.9390879	0.9734907
70	male	Post-	0.9212508	0.9589640	0.9963772
71	male	Post-	0.9394459	0.9791683	1.0155799
72	male	Post-	0.9634720	0.9973786	1.0322316
73	male	Post-	0.9851485	1.0162120	1.0503125
74	male	Post-	1.0047512	1.0353312	1.0714133
75	male	Post-	1.0186554	1.0564956	1.0842223
76	male	Post-	1.0339273	1.0701875	1.1015622
77	male	Post-	1.0505662	1.0865943	1.1212474
78	male	Post-	1.0677691	1.0995526	1.1319463
79	male	Post-	1.0778076	1.1108659	1.1399659
80	male	Post-	1.0849875	1.1174650	1.1527027
81	male	Post-	1.0932975	1.1258090	1.1652680
82	male	Post-	1.0918235	1.1332573	1.1713685
83	male	Post-	1.0937156	1.1379632	1.1747245
84	male	Post-	1.0889706	1.1433632	1.1839858
85	male	Post-	1.0898756	1.1481706	1.1814520
86	male	Post-	1.0951679	1.1482615	1.1906921
87	male	Post-	1.1032524	1.1513952	1.1993125
88	male	Post-	1.1056941	1.1532422	1.1967563
89	male	Post-	1.1183448	1.1553507	1.2033343
90	male	Post-	1.1204613	1.1612646	1.2077006
91	male	Post-	1.1163682	1.1679364	1.2144322

(Continued on Next Page...)

Table S6: Estimated relative change by age and epidemic period *(continued)*

Age	Sex	Period	2.5% Quant.	Median	97.5% Quant.
92	male	Post-	1.1233311	1.1712027	1.2264122
93	male	Post-	1.1238953	1.1775160	1.2325316
94	male	Post-	1.1190689	1.1833409	1.2571602
95	male	Post-	1.1073479	1.1950633	1.2925261
96	male	Post-	1.1065562	1.2082019	1.3304914
97	male	Post-	1.0981154	1.2135210	1.3617853
98	male	Post-	1.0982014	1.2198747	1.4066325
99	male	Post-	1.0856505	1.2299197	1.4652797
100	male	Post-	1.0695956	1.2502039	1.5261327
50	female	Pre-	0.7815025	0.8119882	0.8316218
51	female	Pre-	0.8071039	0.8261550	0.8452974
52	female	Pre-	0.8243696	0.8405848	0.8590363
53	female	Pre-	0.8383646	0.8547825	0.8725730
54	female	Pre-	0.8496527	0.8650251	0.8830974
55	female	Pre-	0.8573498	0.8727603	0.8910158
56	female	Pre-	0.8642901	0.8786580	0.8956651
57	female	Pre-	0.8702012	0.8843515	0.8988677
58	female	Pre-	0.8736729	0.8888036	0.9033350
59	female	Pre-	0.8778217	0.8933030	0.9095484
60	female	Pre-	0.8848391	0.8966094	0.9119570
61	female	Pre-	0.8872541	0.8988580	0.9156757
62	female	Pre-	0.8918710	0.9038785	0.9201624
63	female	Pre-	0.8967339	0.9117880	0.9263951
64	female	Pre-	0.9006645	0.9180494	0.9360093
65	female	Pre-	0.9115157	0.9284884	0.9450184
66	female	Pre-	0.9232702	0.9391268	0.9539645
67	female	Pre-	0.9343386	0.9493078	0.9658324
68	female	Pre-	0.9466225	0.9598666	0.9768599
69	female	Pre-	0.9599895	0.9725636	0.9910834
70	female	Pre-	0.9732785	0.9864411	1.0054248
71	female	Pre-	0.9839807	1.0007139	1.0193345
72	female	Pre-	0.9971176	1.0151275	1.0341489
73	female	Pre-	1.0113780	1.0297433	1.0475144
74	female	Pre-	1.0216369	1.0419215	1.0583797
75	female	Pre-	1.0338095	1.0503043	1.0690208
76	female	Pre-	1.0423978	1.0593416	1.0828863

(Continued on Next Page...)

Table S6: Estimated relative change by age and epidemic period *(continued)*

Age	Sex	Period	2.5% Quant.	Median	97.5% Quant.
77	female	Pre-	1.0550548	1.0721081	1.0931343
78	female	Pre-	1.0629836	1.0798352	1.1013940
79	female	Pre-	1.0701044	1.0872005	1.1107445
80	female	Pre-	1.0759576	1.0949800	1.1196235
81	female	Pre-	1.0809602	1.1015132	1.1283404
82	female	Pre-	1.0863399	1.1076949	1.1310186
83	female	Pre-	1.0887337	1.1100642	1.1292607
84	female	Pre-	1.0834601	1.1041591	1.1254115
85	female	Pre-	1.0750049	1.0992125	1.1225347
86	female	Pre-	1.0646421	1.0926805	1.1197021
87	female	Pre-	1.0678127	1.0850073	1.1139623
88	female	Pre-	1.0571061	1.0840884	1.1142019
89	female	Pre-	1.0552028	1.0846351	1.1160246
90	female	Pre-	1.0594275	1.0859167	1.1127814
91	female	Pre-	1.0614107	1.0890784	1.1149971
92	female	Pre-	1.0540312	1.0917476	1.1144254
93	female	Pre-	1.0496824	1.0903285	1.1205498
94	female	Pre-	1.0453982	1.0905359	1.1295095
95	female	Pre-	1.0389798	1.0900603	1.1392160
96	female	Pre-	1.0351422	1.0895820	1.1490104
97	female	Pre-	1.0054758	1.0883097	1.1553209
98	female	Pre-	0.9873885	1.0854842	1.1639615
99	female	Pre-	0.9540851	1.0856129	1.1827220
100	female	Pre-	0.9254002	1.0769370	1.2069627
50	female	Epi-	0.8706597	0.9865528	1.1377625
51	female	Epi-	0.9103717	1.0079176	1.1309209
52	female	Epi-	0.9374478	1.0193744	1.1285081
53	female	Epi-	0.9657908	1.0372702	1.1249016
54	female	Epi-	0.9968602	1.0535128	1.1181576
55	female	Epi-	1.0172019	1.0706959	1.1240805
56	female	Epi-	1.0422858	1.0882191	1.1361629
57	female	Epi-	1.0590987	1.1033540	1.1588060
58	female	Epi-	1.0696543	1.1158277	1.1677368
59	female	Epi-	1.0866250	1.1326183	1.1731917
60	female	Epi-	1.0950046	1.1436739	1.1897839
61	female	Epi-	1.1079754	1.1597323	1.2046173

(Continued on Next Page...)

Table S6: Estimated relative change by age and epidemic period *(continued)*

Age	Sex	Period	2.5% Quant.	Median	97.5% Quant.
62	female	Epi-	1.1217777	1.1693217	1.2140973
63	female	Epi-	1.1339007	1.1812069	1.2195257
64	female	Epi-	1.1517698	1.1968029	1.2374915
65	female	Epi-	1.1670144	1.2099839	1.2557815
66	female	Epi-	1.1774020	1.2214487	1.2667037
67	female	Epi-	1.1820983	1.2327550	1.2772745
68	female	Epi-	1.1937011	1.2408478	1.2839444
69	female	Epi-	1.1984540	1.2454778	1.2864408
70	female	Epi-	1.2025519	1.2459911	1.2935315
71	female	Epi-	1.1979456	1.2450143	1.2971380
72	female	Epi-	1.1981221	1.2481858	1.3038860
73	female	Epi-	1.1955629	1.2445500	1.2987913
74	female	Epi-	1.1908260	1.2418051	1.2986831
75	female	Epi-	1.1890762	1.2356508	1.2897444
76	female	Epi-	1.1807917	1.2289042	1.2845050
77	female	Epi-	1.1660568	1.2171169	1.2719802
78	female	Epi-	1.1573004	1.2054077	1.2609875
79	female	Epi-	1.1438565	1.1888438	1.2368196
80	female	Epi-	1.1229975	1.1696107	1.2140155
81	female	Epi-	1.0986725	1.1465074	1.1874915
82	female	Epi-	1.0609282	1.1213499	1.1581826
83	female	Epi-	1.0283878	1.0937087	1.1326144
84	female	Epi-	0.9994300	1.0614431	1.1033608
85	female	Epi-	0.9658781	1.0222269	1.0667208
86	female	Epi-	0.9340138	0.9867882	1.0338491
87	female	Epi-	0.8990711	0.9509264	0.9967888
88	female	Epi-	0.8623388	0.9121330	0.9560862
89	female	Epi-	0.8251271	0.8690286	0.9169674
90	female	Epi-	0.7876609	0.8311484	0.8718611
91	female	Epi-	0.7564707	0.7909154	0.8314868
92	female	Epi-	0.7217907	0.7537304	0.8009679
93	female	Epi-	0.6728842	0.7181364	0.7667129
94	female	Epi-	0.6358407	0.6826216	0.7438450
95	female	Epi-	0.5874116	0.6518856	0.7214812
96	female	Epi-	0.5443218	0.6184683	0.7014513
97	female	Epi-	0.5028856	0.5888582	0.6814435

(Continued on Next Page...)

Table S6: Estimated relative change by age and epidemic period *(continued)*

Age	Sex	Period	2.5% Quant.	Median	97.5% Quant.
98	female	Epi-	0.4638019	0.5588165	0.6674853
99	female	Epi-	0.4252971	0.5331682	0.6556862
100	female	Epi-	0.3914726	0.5131565	0.6466017
50	female	Post-	0.8561584	0.9196215	1.0056997
51	female	Post-	0.8696308	0.9157466	0.9794793
52	female	Post-	0.8768128	0.9169513	0.9599675
53	female	Post-	0.8837364	0.9179982	0.9619072
54	female	Post-	0.8900209	0.9170189	0.9605811
55	female	Post-	0.8933345	0.9191002	0.9528291
56	female	Post-	0.8999044	0.9243166	0.9545170
57	female	Post-	0.9056269	0.9267017	0.9537038
58	female	Post-	0.9067890	0.9301585	0.9574895
59	female	Post-	0.9099090	0.9325629	0.9603931
60	female	Post-	0.9103039	0.9340127	0.9650191
61	female	Post-	0.9055331	0.9313394	0.9597812
62	female	Post-	0.9014901	0.9257688	0.9584252
63	female	Post-	0.9007951	0.9246952	0.9600540
64	female	Post-	0.9065868	0.9287671	0.9600954
65	female	Post-	0.9144361	0.9357648	0.9633542
66	female	Post-	0.9240375	0.9425745	0.9707616
67	female	Post-	0.9330892	0.9524920	0.9788020
68	female	Post-	0.9449240	0.9649880	0.9896051
69	female	Post-	0.9564154	0.9773756	1.0043973
70	female	Post-	0.9696848	0.9891217	1.0194564
71	female	Post-	0.9771799	0.9984705	1.0288565
72	female	Post-	0.9883538	1.0094447	1.0396294
73	female	Post-	0.9941772	1.0164871	1.0465611
74	female	Post-	0.9975955	1.0214106	1.0508974
75	female	Post-	1.0046047	1.0268858	1.0513244
76	female	Post-	1.0054259	1.0302623	1.0585851
77	female	Post-	1.0054525	1.0355958	1.0642291
78	female	Post-	1.0086401	1.0388097	1.0640639
79	female	Post-	1.0140248	1.0417650	1.0653672
80	female	Post-	1.0181289	1.0462843	1.0674445
81	female	Post-	1.0208740	1.0492659	1.0715527
82	female	Post-	1.0234667	1.0492613	1.0751581

(Continued on Next Page...)

Table S6: Estimated relative change by age and epidemic period *(continued)*

Age	Sex	Period	2.5% Quant.	Median	97.5% Quant.
83	female	Post-	1.0279543	1.0518600	1.0793775
84	female	Post-	1.0277758	1.0494467	1.0796764
85	female	Post-	1.0202654	1.0496329	1.0780220
86	female	Post-	1.0168089	1.0478910	1.0753413
87	female	Post-	1.0110457	1.0421603	1.0756095
88	female	Post-	1.0045175	1.0395665	1.0735003
89	female	Post-	1.0019788	1.0377520	1.0804643
90	female	Post-	0.9963083	1.0387614	1.0845054
91	female	Post-	0.9978090	1.0405899	1.0900797
92	female	Post-	1.0031960	1.0469077	1.1002063
93	female	Post-	1.0104450	1.0569612	1.1098110
94	female	Post-	1.0183289	1.0652083	1.1371837
95	female	Post-	1.0122996	1.0725546	1.1579867
96	female	Post-	1.0089082	1.0780600	1.1748477
97	female	Post-	0.9980404	1.0853256	1.1877694
98	female	Post-	0.9885380	1.0855871	1.2118314
99	female	Post-	0.9626645	1.0915711	1.2313302
100	female	Post-	0.9308025	1.0983964	1.2661772

Table S7: Estimated relative change by diseases and epidemic period

Disease	Sex	Period	2.5% Quant.	Median	97.5% Quant
Heart attack	male	Pre-	1.4587876	1.4813909	1.4988766
Heart attack	male	Epi-	1.1348368	1.1406969	1.1451895
Heart attack	male	Post-	1.2910142	1.3045119	1.3149078
Hypertension	male	Pre-	1.3289685	1.3441546	1.3604443
Hypertension	male	Epi-	1.1667703	1.1739821	1.1816768
Hypertension	male	Post-	1.2754537	1.2879117	1.3012526
High cholesterol	male	Pre-	1.0890480	1.1023475	1.1150636
High cholesterol	male	Epi-	1.0406806	1.0466022	1.0522285
High cholesterol	male	Post-	1.0723555	1.0830681	1.0932890
Stroke	male	Pre-	1.3269659	1.3482710	1.3739982
Stroke	male	Epi-	1.0055052	1.0058160	1.0061850
Stroke	male	Post-	1.2872210	1.3056506	1.3278639
Diabetes	male	Pre-	1.4113148	1.4311858	1.4567106

(Continued on Next Page...)

Table S7: Estimated relative change by diseases and epidemic period *(continued)*

Disease	Sex	Period	2.5% Quant.	Median	97.5% Quant
Diabetes	male	Epi-	1.1310851	1.1367534	1.1439606
Diabetes	male	Post-	1.2930419	1.3065984	1.3239420
Chronic lung disease	male	Pre-	1.3853012	1.4160244	1.4441781
Chronic lung disease	male	Epi-	1.0221289	1.0236357	1.0249900
Chronic lung disease	male	Post-	1.2716534	1.2923890	1.3112867
Cancer	male	Pre-	1.7504840	1.7790278	1.8104277
Cancer	male	Epi-	1.3190351	1.3296289	1.3411839
Cancer	male	Post-	1.8702604	1.9043950	1.9420201
Stomach, duodenal, peptic ulcer	male	Pre-	1.1589364	1.1860163	1.2118429
Stomach, duodenal, peptic ulcer	male	Epi-	1.0923415	1.1075541	1.1219333
Stomach, duodenal, peptic ulcer	male	Post-	1.2366789	1.2785079	1.3187946
Parkinson	male	Pre-	1.0159426	1.0774327	1.1256920
Parkinson	male	Epi-	1.0145814	1.0706431	1.1144515
Parkinson	male	Post-	1.0144588	1.0700334	1.1134441
Cataracts	male	Pre-	0.9906488	1.0264563	1.0780816
Cataracts	male	Epi-	0.9992595	1.0020610	1.0059455
Cataracts	male	Post-	0.9830489	1.0486636	1.1466133
Hip, femoral fracture	male	Pre-	1.2151848	1.2541101	1.3073349
Hip, femoral fracture	male	Epi-	1.0418899	1.0488299	1.0580485
Hip, femoral fracture	male	Post-	1.2825910	1.3352866	1.4080768
Heart attack	female	Pre-	1.3225599	1.3400570	1.3579099
Heart attack	female	Epi-	1.0176509	1.0184883	1.0193323
Heart attack	female	Post-	1.2811672	1.2961777	1.3114703
Hypertension	female	Pre-	1.2846734	1.2944496	1.3073456
Hypertension	female	Epi-	1.0798183	1.0823307	1.0856248
Hypertension	female	Post-	1.2288441	1.2365316	1.2466568
High cholesterol	female	Pre-	1.1060872	1.1186127	1.1286852
High cholesterol	female	Epi-	1.1312700	1.1469607	1.1596071
High cholesterol	female	Post-	1.0857977	1.0958253	1.1038743
Stroke	female	Pre-	1.2646175	1.2953448	1.3176041
Stroke	female	Epi-	1.0388625	1.0429208	1.0458105
Stroke	female	Post-	1.1868877	1.2078659	1.2229787
Diabetes	female	Pre-	1.3230686	1.3420185	1.3622144
Diabetes	female	Epi-	1.1231901	1.1298381	1.1368629
Diabetes	female	Post-	1.2203246	1.2327305	1.2458965
Chronic lung disease	female	Pre-	1.2968971	1.3176336	1.3386418

(Continued on Next Page...)

Table S7: Estimated relative change by diseases and epidemic period *(continued)*

Disease	Sex	Period	2.5% Quant.	Median	97.5% Quant
Chronic lung disease	female	Epi-	1.0728648	1.0774790	1.0820998
Chronic lung disease	female	Post-	1.2506895	1.2678775	1.2852522
Cancer	female	Pre-	1.5881518	1.6170753	1.6384253
Cancer	female	Epi-	1.1328284	1.1383544	1.1423873
Cancer	female	Post-	1.6654166	1.6988930	1.7236435
Stomach, duodenal, peptic ulcer	female	Pre-	1.1814648	1.2064351	1.2258661
Stomach, duodenal, peptic ulcer	female	Epi-	1.0229706	1.0258887	1.0281235
Stomach, duodenal, peptic ulcer	female	Post-	1.1947126	1.2216702	1.2426735
Parkinson	female	Pre-	0.5195846	0.6686785	0.8667815
Parkinson	female	Epi-	0.6524818	0.7691662	0.9109753
Parkinson	female	Post-	0.4636418	0.6234533	0.8454892
Cataracts	female	Pre-	1.2825368	1.6745456	2.1614218
Cataracts	female	Epi-	1.1208436	1.2666501	1.4238740
Cataracts	female	Post-	1.3211215	1.7805792	2.3692324
Hip, femoral fracture	female	Pre-	1.1725855	1.1984807	1.2371155
Hip, femoral fracture	female	Epi-	1.0211668	1.0241057	1.0283894
Hip, femoral fracture	female	Post-	1.1548769	1.1779192	1.2122096
